# Onepot-Seq: capturing single-cell transcriptomes simultaneously in a continuous medium via transient localization of mRNA

**DOI:** 10.1093/nar/gkac665

**Published:** 2022-08-12

**Authors:** Dongju Shin, Jungwon Choi, Ji Hyun Lee, Duhee Bang

**Affiliations:** Department of Chemistry, Yonsei University, Seoul, Korea; Department of Chemistry, Yonsei University, Seoul, Korea; Department of Clinical Pharmacology and Therapeutics, College of Medicine, Kyung Hee University, Seoul, Korea; Department of Biomedical Science and Technology, Kyung Hee University, Seoul, Korea; Department of Chemistry, Yonsei University, Seoul, Korea

## Abstract

The development of single-cell RNA-seq has broadened the spectrum for biological research by providing a high-resolution analysis of cellular heterogeneity. However, the requirement for sophisticated devices for the compartmentalization of cells has limited its widespread applicability. Here, we develop Onepot-Seq, a device-free method, that harnesses the transient localization of mRNA after lysis to capture single-cell transcriptomes simultaneously in a continuous fluid medium. In mixed-species experiments, we obtained high-quality single-cell profiles. Further, cell type-specific poly(A)-conjugated antibodies allow Onepot-Seq to effectively capture target cells in complex populations. Chemical perturbations to cells can be profiled by Onepot-Seq at single-cell resolution. Onepot-Seq should allow routine transcriptional profiling at single-cell resolution, accelerating clinical and scientific discoveries in many fields of science.

## Graphical Abstract

**Graphical Abstract:**
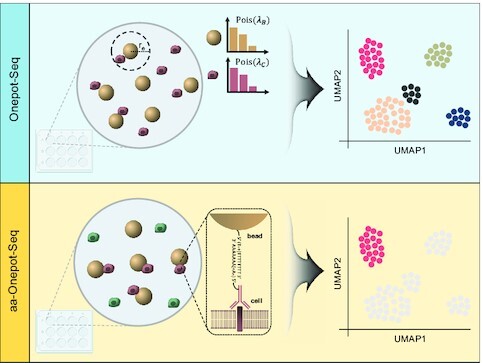
Scheme of Onepot-Seq and aa-Onepot-Seq. Onepot-Seq utilizes transient reaction chambers to capture single-cell transcriptomes in one pot. aa-Onepot-Seq captures single cells of a specific cell type by using cell-type specific poly(A)-conjugated antibodies.

## INTRODUCTION

Single-cell RNA sequencing (scRNA-seq) has been a key tool for unveiling biological heterogeneity and has been considered the gold standard for defining distinct cell states, types and phenotypes for the last decade. Transcriptional profiling of individual cells provides insights into the variations in functional states of heterogeneous populations at an unprecedented resolution (1–3). The versatility of scRNA-seq invites the development of new strategies to easily obtain large numbers of single-cell transcriptomes. However, despite the development of massively parallel approaches, conventional technologies require large sample sizes, laborious processes and expensive devices.

Compartmentalization of single cells has been indispensable for capturing single-cell transcriptomes (4) because each cell should be separated from all others and partitioned into its own reaction chamber, blocking interaction with the outside and allowing each cell to be uniquely barcoded following subsequent lysis. Conventional methods such as manual picking (5,6) and fluorescence-activated cell sorting (FACS) (7–9) allocate one well for each cell as a reaction chamber, limiting the scalability for parallel profiling of single cells. Recently developed massively parallel approaches also involve compartmentalization of single cells. Droplet microfluidics compartmentalizes cells into droplets (10–12), while microwell-based (13,14) approaches physically isolate single cells. The other state-of-the-art technology, combinatorial indexing (15,16), utilizes split-pool barcoding of nucleic acids to uniquely label single cells and requires the isolation of pooled cells in each well.

Physical compartments may not be necessary in single-cell experiments if the contents of the cells can be spatially fixed in a continuous medium. Techniques for capturing single-cell transcriptomes that do not require the physical separation of cells have advantages over conventional single-cell methods. Because the experiments are carried out in a continuous medium, the number of cells can easily be increased with minimal additional costs and time. In addition, more sample was retained because some of the sample is inevitably lost when compartmentalizing the cells. Importantly, no complex devices are needed to compartmentalize cells, expanding the availability of scRNA-seq to many more laboratories. Despite a number of advantages, capturing the single-cell transcriptome without spatial cell separation has been a great challenge due to difficulty in eliminating intercellular mRNA contamination by molecular diffusion.

Here, we developed Onepot-Seq, a novel method for capturing single-cell transcriptomes without physical compartments such as droplets or microwells. Onepot-Seq utilizes a time-constrained, transient reaction chamber for each cell, which is formed around the cell by minimal diffusion of cell contents after gentle lysis. Sparse distribution of both beads and cells results in beads that uniquely capture the transcriptome of a single nearby cell in a continuous fluid medium. This enables simultaneous capture of single-cell transcriptomes in one pot with no dedicated device, greatly simplifying the complex and laborious single-cell RNA sequencing process. We demonstrated that this simple but counterintuitive method has comparable performance with conventional methods and is widely applicable to various single-cell RNA sequencing applications.

## MATERIALS AND METHODS

### Cell lines and cell culture

All cell lines were obtained from the Korean Cell Line Bank (KCLB), except DC2.4, which was a kind gift from Dr Sang-Jun Ha (Yonsei University). All cell lines were maintained at 37°C with 5% CO_2_. Human embryonic kidney HEK293T cells and mouse embryo NIH3T3 fibroblasts were cultured in Dulbecco's modified Eagle's medium (DMEM; Gibco, USA) supplemented with 10% fetal bovine serum (FBS; Gibco, USA) and 1% penicillin–streptomycin (Thermo Fisher Scientific, USA). DC2.4, human acute T cell leukemia, Jurkat, human lung adenocarcinoma NCI-H3122 and NCI-H358, and human colorectal adenocarcinoma SW480 cells were cultured in RPMI 1640 (Gibco, USA) supplemented with 10% FBS and 1% penicillin–streptomycin.

### Poisson modeling of Onepot-Seq

Onepot-Seq assumes that following lysis, mRNAs are locally constrained for a limited time, providing a transient reaction chamber for capturing single-cell transcriptomes. Assuming the radius of the transient reaction chamber is *r*_e_, the number of beads within a reaction chamber is stochastic and is assumed to follow a Poisson distribution:

$$\begin{equation*}Po\ \left( {x;{\lambda }_b} \right) = \frac{{\lambda _b^x{e}^{ - {\lambda }_b}}}{{x!}}\ \end{equation*}$$

where *λ* denotes the average number of beads present in a single transient reaction chamber and *x* denotes the number of beads. Since the beads and the cells are in close contact with the surface, the reaction chamber can be considered a two-dimensional circle.

$$\begin{equation*}{\rm{\ }}{\lambda }_b = {\rho }_b\ \pi r_e^2\end{equation*}$$

where *ρ*_b_ denotes the density of beads on a two-dimensional surface and *r*_e_ denotes the radius of a transient reaction chamber. Cell capture efficiency is calculated as the probability of more than one bead within the reaction chamber:

$$\begin{equation*}P\ \left( {Cell\ yield} \right) = \ 1 - Po\ \left( {x\ = \ 0;{\lambda }_b} \right) = \ 1 - \frac{{\lambda _b^0{e}^{ - {\lambda }_b}}}{{0!}}\end{equation*}$$

$$\begin{equation*}P\ \left( {Cell\;yield} \right) = \ 1 - {e}^{ - {\rho }_b\pi r_e^2}\end{equation*}$$

Note that cell yield is not dependent on cell density, but bead density and the effective capture radius.

Alternatively, we can think of the reaction chamber as a circle around a bead rather than a circle around a cell. In this case, we can assume the number of cells entering within the radius *r* from the center of a bead follows a Poisson distribution:

$$\begin{equation*}Po\ \left( {x;{\lambda }_c} \right) = \frac{{\lambda _c^x{e}^{ - {\lambda }_c}}}{{x!}}\ = \frac{{{{\left( {{\rho }_c\pi r_e^2} \right)}}^x{e}^{ - {\rho }_c\pi r_e^2}}}{{x!}}\ \end{equation*}$$

where *ρ*_c_ is the density of cells on a two-dimensional surface. Then, the probability of multiplets among all input beads is

$$\begin{equation*}P ( {multiplet} ) = \ 1 - Po ( {x \le 1;{\lambda }_c} ) = 1 - \frac{{\lambda _c^0{e}^{ - {\lambda }_c}}}{{0!}} - \frac{{\lambda _c^1{e}^{ - {\lambda }_c}}}{{1!}}\end{equation*}$$

We can calculate the probability of beads being barcoded with selective amplification as follows:

$$\begin{equation*}P\ \left( {bead\ yield} \right) = \ 1 - Po\ \left( {x\ = \ 0;{\lambda }_c} \right) = \ 1 - \frac{{\lambda _c^0{e}^{ - {\lambda }_c}}}{{0!}}\end{equation*}$$

Therefore, the probability of multiplets, given that cells are barcoded with selective amplification, is given by the equation:

$$\begin{equation*}P\ \left( {\begin{array}{@{}*{1}{c}@{}} {multiplet\ in}\\ {barcoded\ bead} \end{array}} \right) = \frac{{1 - {e}^{ - {\lambda }_c}\left( {1 + {\lambda }_c} \right)}}{{1 - {e}^{ - {\lambda }_c}}}\ = \ 1 - \frac{{{\rho }_c\pi r_e^2{e}^{ - {\rho }_c\pi r_e^2}}}{{1 - {e}^{ - {\rho }_c\pi r_e^2}}}\end{equation*}$$

We used the equations above to calculate the theoretical values for multiplets and capture rates at various cell densities.

### Onepot-Seq: library preparation for sequencing

Cells were harvested using standard cell culture methods. After removing the medium from the culture flasks, adherent cells were washed once with 1× phosphate-buffered saline (PBS) and trypsinized in 2 ml of trypsin (Trypsin-EDTA; Gibco, USA) for 3–10 min at 37°C and the trypsin was inactivated with 8 ml of growth medium. Cells were washed three times with PBS-BSA (0.1% bovine serum albumin in 1× PBS) and resuspended in 10 ml of PBS-BSA to yield a concentration in a range of 500–1000 cells in 1 μl. Cells were passed through a 40 μm filter and counted using a hemocytometer. Barcoded beads used in Onepot-Seq were purchased from Chemgene [cat. No. MACOSKO-2011-10(V+)]. Beads for Onepot-Seq were washed three times with PBS-BSA and resuspended in incubation buffer [6% Ficoll PM-400 (GE Healthcare), 20 mM EDTA (Biosesang), 200 mM Tris pH 7.5 (Biosesang)] at a concentration of 20 000 beads in 100 μl.

After beads and cells were prepared, 900 μl of incubation buffer was added to a well of a 12-well plate (TPP®, Switzerland) and 100 μl of prepared beads (20 000 beads in 100 μl of incubation buffer) were added to the well. Then, prepared cells were added to the well at the desired concentration. The volume of added cells was 1–10 μl to avoid significantly increasing the total volume. The plate was rocked gently three times vertically and three times horizontally to evenly spread the beads and cells. It is important to avoid a circular motion when rocking because it results in the clumping of cells and beads in the middle of the plate. Without disturbing the position, the plate was incubated for 15 min to settle the beads and cells to the bottom of the plate.

Cell lysis depended on the method. In the diffusive lysis method, which is the standard protocol of Onepot-Seq, 200 μl of lysis buffer A [1:1 mix of 20% *N*-lauroylsarcosine sodium salt solution (Sigma) and incubation buffer] was added to the well. When adding the lysis buffer, the edge of the pipette tip was aligned with the water surface so that the flow was as low as possible, and the buffer was slowly added in a circular motion to spread it evenly. The plate was then incubated for 15 min to lyse the cells and allow the beads to capture the mRNA. Since the incubation buffer is denser than lysis buffer A, lysis buffer A sits atop the cell–bead solution and diffuses slowly to the bottom of the well. Normally, cells began to lyse ∼5 min after adding the buffer, most cells were lysed at 10 min and complete lysis took 15 min. In the direct lysis method, 1000 μl of lysis buffer B [incubation buffer supplemented with 0.2% *N*-lauroylsarcosine sodium salt solution and 50 mM dithiothreitol (DTT; Biosesang)] was added directly to cells that were suspended in PBS-BSA instead of incubation buffer. In this lysis method, 1 ml of lysis buffer B was added along the side of the well. Since the density of PBS-BSA is lower than that of lysis buffer B, the lysis buffer directly sinks to the bottom of the well, lysing cells within 100 s. The above lysis steps were performed at 4°C.

After lysis, beads were kicked up by adding 1 ml of 6× SSC (Biosesang, Korea) and 2 ml of the solution was immediately transferred to 30 ml of ice-cold 6× SSC in a Falcon tube. To maximize bead recovery, the remaining beads were recovered by adding another 1 ml of 6× SSC, pipetting and transferring them to the first Falcon tube. The beads were centrifuged at 1000 × *g* for 1 min and washed twice with 1 ml of 6× SSC, then once with 50 μl of Maxima 5X RT buffer (Thermo Fisher Scientific, USA). Reverse transcription, exonuclease treatment and cDNA amplification were performed as described in the Drop-Seq protocol instructions (10) with some modifications. Briefly, beads were washed once with TE-SDS [TE buffer (Biosesang) supplemented with 0.5% SDS (Biosesang)] and twice with TE-TW [TE buffer, supplemented with 0.01% Tween-20 (Sigma)], and once with 10 mM Tris pH 8.0 (Biosesang) after the reverse transcription reaction (30 min at 25°C and 90 min at 42°C). Then, beads were washed once with TE-SDS, twice with TE-TW and twice with nuclease-free water (Invitrogen) after exonuclease activity reaction (45 min at 37°C).

The cDNA was divided into eight tubes per single Onepot-Seq well reaction and amplified using the KAPA HiFi HotStart PCR Kit (Kapa Biosystems Inc., Switzerland). cDNA amplification was performed in a 50 μl polymerase chain reaction (PCR), which included 4 μl of 10 μM SMART PCR primer (5′-AAGCAGTGGTATCAACGCAGAGT-3′) (Integrated DNA Technologies), 25 μl of KAPA HiFi DNA polymerase and up to 21 μl of nuclease-free water. PCR was performed using the following protocol: 3 min at 95°C; four cycles of 20 s at 98°C, 45 s at 65°C, 3 min at 72°C; nine cycles of 20 s at 98°C, 20 s at 67°C, 3 min at 72°C; and 5 min at 72°C. The PCR products were pooled and purified twice using 0.6× AMPure (Beckman Coulter, USA) beads according to the manufacturer's instructions. The cDNA products were fragmented and further amplified using the Nextera XT DNA Library Preparation Kit (Illumina, USA).

The detailed step-by-step protocol for Onepot-Seq is provided in protocol.io (https://www.protocols.io/view/onepot-seq-b5u3q6yn).

### Antibody-assisted Onepot-Seq (aa-Onepot-Seq)

Cells were harvested using standard cell culture methods described above. Up to 1.0 × 10^6^ cells were gently suspended in 100 μl of Cell Hashing Staining buffer (Cat. No. #420201; BioLegend, USA) containing 2 μl of appropriate TotalSeq antibody (BioLegend, USA) and incubated for 30 min at 4°C. Cells were washed twice with PBS-BSA, filtered through a 40 μm filter and counted. After washing the beads as described above, 20 000 beads were incubated with an appropriate number of cells for 1 h in 100 μl of PBS-BSA in a well of a 96-well plate to maximize cell–bead contact. The appropriate number of input cells depends on the binding efficiency of the beads and cells, which is affected by the level of surface expression of the target protein and the antibody affinity. After incubation, without disturbing the cell–bead binding, cells and beads were carefully transferred to a well of a 12-well plate containing 900 μl of incubation buffer (for the depletion of unbound cells at this step, see below). After gently rocking the plate to spread the beads and cells evenly, cell lysis, library preparation and sequencing were performed as described above for Onepot-Seq. A step-by-step protocol for aa-Onepot-Seq is provided in protocol.io (https://www.protocols.io/view/aa-onepot-seq-b7mtrk6n).

### Depletion of unbound cells by a cell strainer

Depletion of unbound cells was performed using a 20 μm pluriStrainer (pluriSelect, Germany). After the incubation of beads and cells, the solution containing the cell–bead complex was transferred to the pluriStrainer on the Connector Ring (pluriSelect, Germany) assembled with a 50 ml Falcon tube. The solution was forced to flow using a syringe by pulling the piston with gentle pressure. To remove unbound cells, 1 ml of PBS-BSA was added to the pluriStrainer and washed away using the syringe. After three washes, the pluriStrainer was removed, inverted and placed onto another 50 ml Falcon tube, and 1 ml of PBS-BSA was slowly added to recover the cell–bead sample from the pluriStrainer. Samples were then transferred to a well of a 12-well plate and spread evenly. Cell lysis, library preparation and sequencing were performed as described above.

### Theoretical modeling of mRNA diffusion after release from a cell

Assuming radially symmetrical diffusion in spherical coordinates, the diffusion equation for mRNA after cell lysis is given as follows:

$$\begin{equation*}\frac{1}{D}\ \frac{{\partial c\left( {r,t} \right)}}{{\partial t}} = \left( {\frac{{{\partial }^2}}{{\partial {r}^2}} + \frac{2}{r}\frac{\partial }{{\partial r}}} \right)\ c\left( {r,t} \right)\end{equation*}$$

where *D* is the diffusion coefficient of mRNA, *c* is the concentration of mRNA, *r* is the distance from the cell and *t* is time. Assuming that a cell is a sphere of radius *R* and the concentration of mRNA within the cell is homogeneous, the solution to the above equation gives the concentration profile over time and distance (17):

$$\begin{equation*}c\ \left( {r,t} \right) = \frac{{{c}_0}}{{2r\sqrt {\pi Dt} }}\ \mathop \smallint \limits_0^R \xi \left\{ {\exp \left[ { - \frac{{{{\left( {r - \xi } \right)}}^2}}{{4Dt}}} \right] - \exp \left[ { - \frac{{{{\left( {r + \xi } \right)}}^2}}{{4Dt}}} \right]} \right\}{\rm{d}}\xi \end{equation*}$$

where *c*_0_ is the initial concentration of intracellular mRNA and *R* is the radius of a cell. The diffusion coefficient of mRNA was estimated to be 1.0 μm^2^/s based on the diffusion coefficients of DNA fragments in water or cytosol from the literature (18–21). The cell size and the initial concentration of mRNA were estimated to be 5 μm and 1.2 μM, respectively. mRNA concentration in the droplets of Drop-Seq was calculated by diluting the mRNA of a cell by the volume of a droplet. We used the integrate() function in the ‘stats’ package of R to integrate the above equation to yield the mRNA concentration–distance profile. The diffusion distance was calculated as the distance from the center of the cell where 90% of mRNA is contained. We used the uniroot() function in the ‘stats’ package of R to find a root for the equation of diffusion distance using the following arguments: lower = 1E-4, upper = 1E4, tol = 1E-10.

### Onepot-Seq optimization for lysis time and cell density

To optimize the Onepot-Seq protocol, several conditions of the cell lysis time and the cell density were tested. Overall procedures were performed as described above for Onepot-Seq. Briefly, HEK293T and NIH3T3 cells were prepared for the human–mouse mixed-species experiment. To confirm the cell density condition, different numbers of cells (1000, 2000, 4000, 8000, 12 000 and 20 000) in each well of a 12-well plate were tested. To confirm the cell lysis condition, different lysis times (100 s, 5 min, 15 min, 30 min and 60 min) were tested by both the diffusive lysis and the direct lysis methods.

### Comparison with Drop-Seq

Drop-Seq was performed as previously described (10). Briefly, DC2.4 and Jurkat cells were seeded in T75 flasks at a density of 1.0 × 10^6^ cells per flask. After 3 days of culture, DC2.4 and Jurkat cells were harvested as described above. Cells were washed three times with PBS-BSA, passed through a 40 μm filter, counted using a hemocytometer and then pooled in equal amounts. Cells were diluted to 100 cell/μl in PBS-BSA and Drop-Seq was performed using a bead concentration of 1.0 × 10^6^ cell/ml. For comparing Onepot-Seq with Drop-Seq, HEK293T and NIH3T3 cells at a density of 1000 cells per well in a 12-well plate were used in Onepot-Seq. Because of large differences in size among human cells, only mouse cells were used for this comparison. The diffusive lysis, library preparation and sequencing were performed as described above. cDNA traces of Onepot-Seq and Drop-Seq libraries and the percentages of genome-mapped reads mapped to coding regions, untranslated regions, introns and intergenic regions are shown in Supplementary Figure S15A and B, respectively.

After alignment, bam files were randomly subsampled for the aligned reads from 10% to 100% at intervals of 10%. Each bam file was subjected to the same processing pipeline as described above. Then, at each sequencing depth, we obtained the average raw sequencing depth and average UMIs (unique molecular identifiers) of cell barcodes with > 500 transcripts from the original data.

Assuming that the average UMI will be saturated along the sequencing depth, fitting and predicting the saturation level of genes and UMIs were performed using the following equation:

$$\begin{equation*}y\ = \ a + \frac{b}{{x + c}}\end{equation*}$$

where *x* is the sequencing depth, *y* is the average number of UMIs or genes and *a*, *b* and *c* are fitting parameters.

### The effect of poly(A)-conjugated antibody labeling

To examine the effect of poly(A)-conjugated antibody labeling, Jurkat and DC2.4 cells were labeled with anti-human Hashtag1 TotalSeq antibody (BioLegend, Cat. No. #394601) and anti-mouse Hashtag1 TotalSeq antibody (Cat. No. #155801), respectively. Equal amounts of Jurkat and DC2.4 cells were prepared for the human–mouse mixed-species experiment. Different numbers of cells (1000, 2000, 4000 and 800) were incubated with beads to form bead–cell complexes in each well of a 96-well plate. Bead–cell complexes were transferred to a well of a 12-well plate. The direct lysis, library preparation and sequencing were performed as described above. For confocal microscopy images, Jurkat and DC2.4 cells were stained with CellTrace™ CFSE (CFSE; Thermo Fisher Scientific, USA) and CellTrace™ Far Red (CTFR; Thermo Fisher Scientific, USA) according to the manufacturer's instructions. Then, cells were labeled in three ways: (i) labeled with anti-human; (ii) labeled with both anti-human and mouse antibodies; and (iii) labeled with no antibody (control). Samples were then incubated with beads before imaging.

### Selective capture in human–mouse mixed population using human- or mouse-specific antibodies

Jurkat and DC2.4 cells were prepared and mixed in equal amounts. Three samples were labeled using only anti-human antibody, anti-mouse antibody and both anti-human and mouse antibodies, respectively. Following labeling, unbound cells were depleted using the strainer as described above. After sufficient washing, bead–cell complexes were recovered from the strainer and transferred to a well of a 12-well plate. The direct lysis, library preparation and sequencing were performed as described above.

### Flow cytometry

Jurkat cells were harvested using standard cell culture methods. Briefly, after 24 h of culture, cells were harvested, washed twice with PBS and stained with CellTrace™ Violet (CTV; Thermo Fisher Scientific, USA) according to the manufacturer's protocol. Briefly, 5.0 × 10^6^ cells were pelleted and incubated with 1 ml of CTV solution (1:1000 dilution) for 20 min at 37°C with protection from light. Then, 4 ml of complete culture medium were added and the cells were incubated for 5 min at 37°C. A total of 1.0 × 10^6^ cells were pelleted, resuspended in a solution containing 2 μl of anti-human Hashtag1 TotalSeq antibody (Cat. No. #394601; BioLegend, USA) in 100 μl of staining buffer and incubated for 30 min at 4°C. After incubation, cells were washed twice with PBS-BSA and once with FACS buffer (2% FBS in 1× PBS), resuspended in FACS buffer, passed through a 40 μm filter and counted using a LUNA-II™ automated cell counter (Logos Biosystems, Korea) and cell-counting slides (Logos Biosystems, Korea). Beads were washed and prepared as previously described. Lastly, 2000 cells were incubated with 20 000 washed beads in a 1.5 ml tube for various times at room temperature. Immediately after incubation, the fraction of bead–cell multiplets was measured and analyzed by flow cytometry on a BD FACS Aria II (BD Biosciences, USA). Quantification and downstream analysis were performed using FlowJo10 (FlowJo.LLC).

### PBMC isolation from blood

Human peripheral blood mononuclear cells (PBMCs) were obtained from three healthy donors with approval of the Institutional Review Board (IRB) of Yonsei University (7001988-202106-HR-864-04). Blood from healthy donors was collected in Cell-Free DNA BCT tubes (STRECK). PBMCs were isolated according to standard procedures by density gradient centrifugation using Histopaque medium (Sigma, USA) and washed with PBS (Gibco, USA). To reduce platelet contamination, five additional low-speed centrifugation (10 min at 120 × *g*) steps were performed with the centrifuge brake off. Cells were resuspended in PBS-BSA and counted for subsequent use in experiments.

### Profiling various cell types in PBMCs by aa-Onepot-Seq using various antibodies

Overall procedures were performed as described above for aa-Onepot-Seq. Human PBMCs, which are isolated from blood, were labeled with appropriate TotalSeq antibody [BioLegend, Cat. No. #300475(anti-human CD3), #300563(anti-human CD4), #301067(anti-human CD8), #301855(anti-human CD14), #302061(anti-human CD16), #302259(anti-human CD19)]. The number of input cells was varied for each antibody according to the composition of the cell type targeted by the antibody in PBMCs. The depletion of unbound cells was performed to increase the selectivity. After depletion, cell spreading, cell lysis by the direct lysis method, library preparation and sequencing were performed as described above for Onepot-Seq.

### Enrichment and sequencing of CTCs in PBMCs with aa-Onepot-Seq

To mimic circulating tumor cells (CTCs) in PBMCs of patients with advanced tumors, we spiked tumor cell lines to PBMCs from healthy donors. Overall procedures were performed as described above for aa-Onepot-Seq. NCI-H3122 was spiked to 1.0 × 10^6^ PBMCs at two different ratios (1% and 0.1%). Cells were then labeled with anti-human CD326/Ep-CAM TotalSeq antibody (Cat. No. #324241) and projected to aa-Onepot-Seq.

### KRAS(G12C) inhibitor treatment of cell lines with different KRAS mutation status

Single-cell expression profiling of cells perturbed by a KRAS(G12C) inhibitor was performed as follows: NCI-H3122, NCI-H358 and SW480, with KRAS genotypes of wild type, G12C, and G12V, respectively, were trypsinized after 24 h of culture. Then, cells were washed with PBS, and plated in poly-d-lysine-coated wells of a 12-well plate at a density of 1000 cells per well in 1 ml of medium. Four wells per cell line were seeded for different drug concentrations, the cells were evenly distributed by rocking the plate and the plate was incubated at 37°C for 8 h to allow attachment. After incubation, 700 μl of the medium was removed and another 700 μl of medium containing various concentrations of AMG-510 (Selleckchem, Cat. No. S8830) was added to the wells while minimizing any disturbance of the cells adhering to the bottom. After 6 h of drug treatment at 37°C, 700 μl of the medium was removed and 700 μl of the incubation buffer containing 20 000 beads was added to the well. The beads were spread evenly by rocking the plate and incubated for 15 min to allow them to settle. Cells were lysed by the diffusive lysis method and the preparation of single-cell libraries was performed as described above.

### Data processing, read alignment and construction of gene expression matrix

Read alignment was performed as in Macosko *et al.* (10). Briefly, for each NextSeq sequencing run, raw sequencing data were converted to FASTQ files using bcl2fastq2 (Illumina, USA). Raw sequencing data were demultiplexed by Nextera N7xx indices corresponding to individual samples. Reads were first tagged according to the 12 bp cell barcode sequence and the 8 bp UMI in ‘read 1.’ Then, reads in ‘read 2’ were aligned with the hg19 or hg19-mm10 concatenated reference using the STAR aligner, depending on the experiments, and collapsed onto 12 bp cell barcodes that corresponded to individual beads. Barcodes were collapsed with a single-base error tolerance (Hamming distance = 1), with additional provisions for single insertions or deletions. To obtain quantitative counts of individual transcripts, UMIs were also collapsed with a single-base error tolerance (Hamming distance = 1). A digital expression matrix was obtained by collapsing filtered and mapped reads for each gene by the UMI sequence within each cell barcode.

### Analysis of the human–mouse mixed-species experiment

After aligning the reads with the hg19-mm10 concatenated reference using the STAR aligner and collapsing the barcodes, digital expression matrices of human and mouse cells were obtained by separating human and mouse aligned reads, respectively. The number of human and mouse transcripts was calculated for each cell barcode, and a barcode with > 500 transcripts was defined as a cell. The ratio of human transcripts within a cell barcode was calculated, and cell barcodes with a human transcript ratio > 0.9 were defined as human cells, < 0.1 as mouse cells and the rest as mixed species. The greater of the human transcript ratio and the mouse transcript ratio within a cell was defined as the species purity of the cell. The multiplet rate was calculated as the proportion of mixed species in total cells and the cell yield was calculated as the ratio of the number of cells finally obtained from sequencing versus the number of input cells.

### Analysis of human PBMCs

Following the alignment of the sequencing reads, downstream analysis of the PBMC experiment was performed using the R package ‘Seurat’ (22). Low-quality and dead cells were filtered out by determining the mitochondrial read fraction, the number of UMIs and the number of genes. After quality filtering, we obtained 3927 cells with > 300 genes. The RNA expression matrix was log-normalized, centered, scaled and processed for further analysis. To cluster single cells, we ran principal components analysis (PCA) using the expression matrix of variable genes and then performed Uniform Manifold Approximation and Projection (UMAP) (23) using the top 15 PCA components. We identified seven clusters using the FindClusters function in Seurat with ‘res’ set to 0.5. Among the seven clusters, we removed cells in cluster 6 which represented low-quality cells as identified by co-expression of multiple cell type-specific markers. To find markers for each cluster, the FindAllMarkers function was used with the parameters set as follows: min.pct = 0.25, logfc.threshold = 0.25. The clusters were assigned to cell types based on the expression of known markers for major PBMC types (24). Normalized and scaled gene expression data were used for the heatmap. The fold change for the frequency of each cell type in each sample was calculated based on the composition of the ‘Hash’ sample from each donor. Enrichment for NCI-H3122 cells was calculated based on their original frequency.

### Analysis of the dose-dependent KRAS(G12C) inhibitor experiment

For cells treated with the KRAS(G12C) inhibitor, following sequence alignment and quality filtering using Seurat, we identified a total of 3949 single cells in which at least 1000 genes were detected. After normalizing and scaling the expression matrix, we reduced the dimensionality of our dataset by running PCA using the expression matrix with 2000 highly variable genes. We then selected the first 11 PCA components and performed *t*-distributed stochastic neighbor embedding (*t*-SNE). Three clusters were identified, and each cluster was assigned to each input cell line based on the sample identities, cell line-specific expression markers and single nucleotide polymorphisms (SNPs). Expression markers for each cell line were identified using the FindAllMarkers function in Seurat with min.pct = 0.25 and logfc.threshold = 0.25. We used scaled single-cell expression of the top 10 markers for each cell line for the expression heatmap. Classification of the cell cycle phase was performed with the CellCycleScoring function in Seurat. We classified each single cell to the G2–M, S or G1 phase using the cell cycle markers reported by Tirosh *et al.* (25).

### SNP-based cell line classification

We used demuxlet (26) to determine whether single-cell libraries generated by Onepot-Seq could identify cell lines with SNPs. Briefly, for generating input vcf files, we downloaded vcf files of single nucleotide variants identified by Caveman (27) for each cell line from the cell line project of COSMIC (Catalogue Of Somatic Mutations In Cancer) database and merged them to obtain 33 425 SNPs of a single vcf file. Then, we used Freebayes to estimate allelic fractions for each cell line across the reference SNP panel using bulk averaged single-cell data. The following commands were used when running Freebayes: –pooled-continuous, –report-monomorphic and –only-use-input-alleles. A merged vcf file with allelic counts for each cell line was obtained and we ran demuxlet using a bam file aligned with STAR and tagged with a cell barcode and UMI as an input with commands –field GT –alpha 0 –alpha 0.5.

### Comparison of gene expression with a cell line expression database

To confirm the accuracy of the cell line classifications, we compared our single-cell dataset with the gene expression database of cell lines from the CCLE project (21Q1 DepMap gene expression data). For this analysis, we averaged our untreated control single-cell data to obtain bulk expression data for each cell line and calculated the Pearson correlation with CCLE datasets.

### Differential expression analysis

To identify differentially expressed genes (DEGs) due to AMG-510 drug treatment, we performed various tests for DEG identification per dose per cell line compared with untreated control cells using the FindMarkers function in Seurat with min.pct = 0.1 and logfc.threshold = 0.1. Differential gene expression test results and statistics, with adjusted *P* < 0.05 using a likelihood-ratio test, Wilcoxon rank-sum test, negative binomial generalized linear model and Poisson generalized linear model, are listed in Supplementary Table S1.

### Gene set enrichment analysis

For analysis of gene set enrichment of transcriptional response signatures, we used the R package ‘msigdbr’ (28,29). Briefly, we used Fisher's exact test to measure the set overlap between each gene set and the 50 top up-regulated and down-regulated genes based on the absolute fold change with an adjusted *P* < 0.05 and a log fold change > 0.5. The *P*-value was obtained using the likelihood-ratio test and the gene sets used in the analysis were a combination of the ‘Hallmark’ and ‘Canonical’ gene set collections from MSigDB v7.2 (28,29). The results of the gene set enrichment analysis for AMG-510 treatment per dose per cell line are listed in Supplementary Table S2.

### Pseudotime analysis

For psuedotime analysis of KRAS(G12C) treatment in the H358 cell line, we applied the R package ‘Monocle3’ (30). A single-cell dataset for the H358 cell line was extracted from the analysis of the KRAS(G12C) inhibitor treatment experiment described above using Seurat. The data were normalized and pre-processed before further analysis. To remove the cell cycle effect on the cell trajectory, we mutually aligned cells with groups of different cell cycle states using the align_cds function. We reduced the dimension and clustered cells with a UMAP algorithm. After fitting a principal graph to the data using the learn_graph function, we ordered cells along the trajectory by defining the node closest to untreated cells as the origin and calculated pseudotime for each single cell. To identify genes that were differentially expressed across the single-cell trajectory, we performed Moran's I test that measures multidirectional and multidimensional spatial autocorrelation. Genes with a *q*-value < 0.01 were selected as DEGs. For the top 24 DEGs, in descending order of *q*-value, gene expression patterns as a function of pseudotime are shown in Supplementary Figure S14E.

### KRAS(G12C) output score determination

We determined KRAS(G12C)-induced and KRAS(G12C)-suppressed scores for each single cell as previously described by Xue *et al.* (31). Briefly, lists of 563 induced and 447 suppressed genes were obtained from the KRAS(G12C) inhibitor treatment of bulk RNA-seq experiment by Xue *et al.* We filtered genes with average log count of < 0.1 from our dataset to remove genes with undetected or low expression. The remaining genes were normalized across all cells in the dataset. The KRAS(G12C)-induced and -suppressed score in each single cell was calculated by averaging the normalized expression values from genes that were, induced or suppressed by the KRAS(G12C) inhibitor, respectively.

## RESULTS

### Overview of Onepot-Seq

A key idea of Onepot-Seq is that cellular mRNA, given its low diffusivity, can be locally confined for a limited time after cell lysis. Thus, it can be captured by nearby barcoded beads containing poly(dT) oligonucleotides, cell barcodes, a universal primer sequence and a UMI. It represents a transient spherical reaction chamber with a radius *r*_e_ around the cell, and only those beads within the radius of this reaction chamber can capture the mRNA of the nearby cell. We hypothesized that given the rapid decrease in mRNA concentration with distance, if *r*_e_ is far less than the distance between sparsely distributed cells the beads can capture the unique profiles of single cells. The effective capture radius, *r*_e_, is determined by the diffusion rate of mRNAs in solution and the time since the mRNA was released from the cell. For randomly distributed cells and beads, the number of cells within the radius *r*_e_ of the beads can be assumed to follow a Poisson distribution. Cell capture efficiency and doublet rate can also be calculated (see the Materials and Methods).

**Figure 1.**
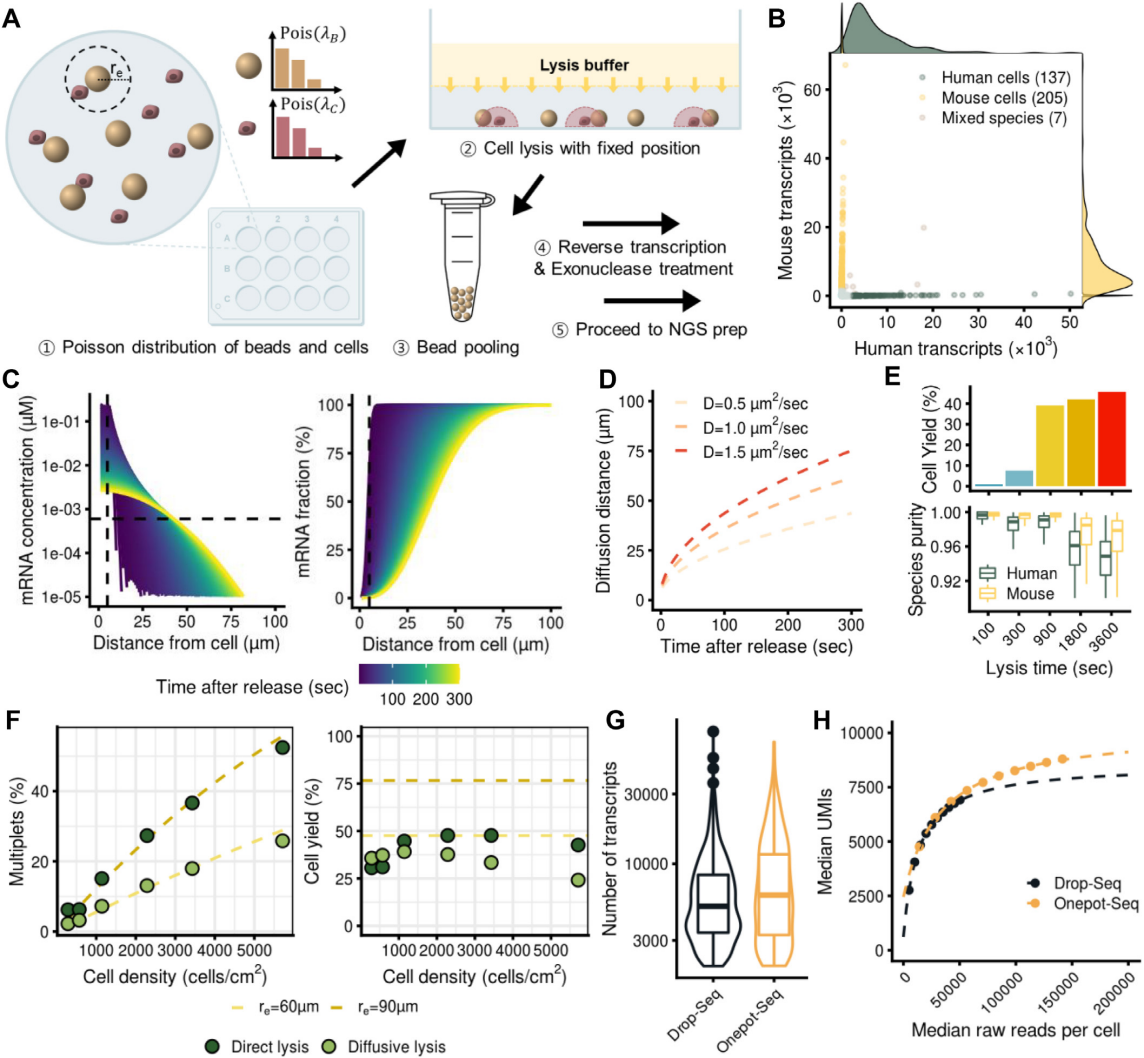
Scheme and optimization experiments for Onepot-Seq. (**A**) Scheme of Onepot-Seq: (1) single cells are positioned in a well with a Poisson distribution. (2) Cells were lysed by the diffusion of lysis buffer from above and through the incubation buffer. (3) Beads were recovered after lysis and pooled. (4) Reverse transcription and exonuclease treatment were performed on the pooled beads. (5) Libraries were processed for scRNA-seq. (**B**) A scatter plot showing the number of UMIs aligned to the human or mouse genome. A cell density of 1000 cells per well in a 12-well plate was used. (**C**) Distance profiles for changes in mRNA concentration over time after mRNA release (left). Profiles of cumulative mRNA release versus distance over time (right). (**D**) Theoretical values for the diffusion distance comprising 90% of mRNA versus time for each diffusion coefficient. (**E**) Cell yield versus lysis time (top). Species purity versus lysis time (bottom). (**F**) Changes in the rate of multiplets (left) and cell yield (right) at various cell densities and different lysis conditions. The dashed lines indicate the theoretical values calculated assuming a Poisson distribution of beads and cells. (**G**) Number of transcripts detected in single-cell libraries by Drop-Seq and Onepot-Seq. (**H**) Median number of UMIs at various sequencing depths for Drop-Seq or Onepot-Seq.

Onepot-Seq requires the spatially stochastic positioning of cells and beads in fixed locations (Figure 1A; Supplementary Figure S1) which is achieved just by pipetting cells and beads onto a specific surface area (e.g. a single well). The distribution of the cells and beads can be easily controlled by adjusting their concentrations in solution. Then, without disturbing their positions, cells are lysed by the diffusion of a detergent. After lysis, the beads are carefully recovered and subjected to reverse transcription, exonuclease treatment and amplification of the library as previously described (10).

### Optimizing experimental conditions for Onepot-Seq

To demonstrate the theoretical feasibility of Onepot-Seq and to assess the *r*_e_, we simulated diffusion of mRNA released immediately after lysis from a cell using several parameters in the literature (18–21) (see the Materials and Methods) (Figure 1C, D). We observed that the concentration of mRNA decreases exponentially with distance, and most of the mRNA is localized within a few tens of micrometers of the cell. By 5 min after mRNA release from the cell, the concentration of mRNA within a 40 μm radius around the cell was higher than that of Drop-Seq (10) in which mRNA of a cell is dissolved evenly in a 1 nl droplet (Figure 1C). Further, 90% of the total mRNA was localized within a 60 μm radius around the cell after 5 min (Figure 1D). Thus, mRNAs released from a cell are highly localized within a few minutes at a sufficiently high concentration to be captured by nearby beads. This suggests the possibility of simultaneous capture of multiple single-cell transcriptomes in a continuous fluid medium.

To determine the optimal lysis condition, we mixed human and mouse cell lines and varied the lysis time (Figure 1E). Shallow sequencing of Onepot-Seq libraries revealed that the cell yield was nearly saturated by 15 min of lysis (Figure 1E, top), while the species purity for single cells remained high (Figure 1E, bottom). Lysis times of 100 s and 5 min showed relatively low cell yield and a high fraction of ambient transcripts in cumulative curves, indicating insufficient lysis and low-quality libraries **(**Supplementary Figure S2). After 30 min of lysis, cell yield increased slightly, while the species purity began to decrease due to cross-contamination from other cells in the vicinity. Thus, to maximize the cell yield, the quality of libraries and the resolution to a single cell, we lysed cells for 15 min in subsequent experiments. Note that the lysis time was the time after adding the lysis buffer, which is different from the time after mRNA was released from the cell because of the time it takes for the detergent to diffuse to the bottom.

We next examined the effect of cell density on Onepot-Seq (Figure 1F; Supplmentary Figure S3). The process by which cells enter the effective capture radius of a bead is stochastic and can be assumed to follow a Poisson process. Thus, multiplet rates, at which two or more cells enter a bead's transient reaction chamber, are a function of the cell density on a two-dimensional plane and the effective capture radius of the transient reaction chamber (see the Materials and Methods). We estimated *r*_e_ by fitting the experimental data to the Poisson modeled equation in experiments using either (i) direct lysis where the lysis buffer is added directly to the cells or (ii) diffusive lysis where the lysis buffer is slowly added to the incubation buffer and detergent diffuses to the cells (see the Materials and Methods). Experiments with direct lysis matched well with a theoretical curve of *r* = 90 μm, while the diffusive lysis method conformed to a curve of *r* = 60 μm, suggesting an additional contribution of convection to diffusion in the direct lysis method **(**Figure 1F, left). Cell yield does not depend on cell density but rather on bead density. As expected, both direct lysis and diffusive lysis showed invariant cell yield with cell densities (Figure 1F, right). Absolute values of cell yield did not agree with the theoretical values, indicating some loss of cells by the filtering out of cells with low-quality transcripts or low levels of transcripts.

Deep sequencing of a mixed human–mouse population with diffusive lysis and with a cell density of 1000 cells per well in a 12-well plate yielded high-quality single-cell profiles of 137 human cells and 205 mouse cells with a multiplet rate of 2.0% (Figure 1B). To compare the capture efficiency with another scRNA-seq method, we performed a Drop-Seq experiment with identical mRNA capture beads and observed comparable numbers of captured genes and transcripts (Figure 1G). In our Drop-Seq experiment, the median number of transcripts captured per cell was 5127, and the median number of genes identified per cell was 2257, whereas in Onepot-Seq, the median numbers of transcripts and genes captured per cell were 6110 and 2364, respectively. Saturation analysis of UMIs with subsampled data was also comparable between Onepot-Seq and Drop-Seq (Figure 1H).

### Application of cell type-specific antibodies in Onepot-Seq

**Figure 2.**
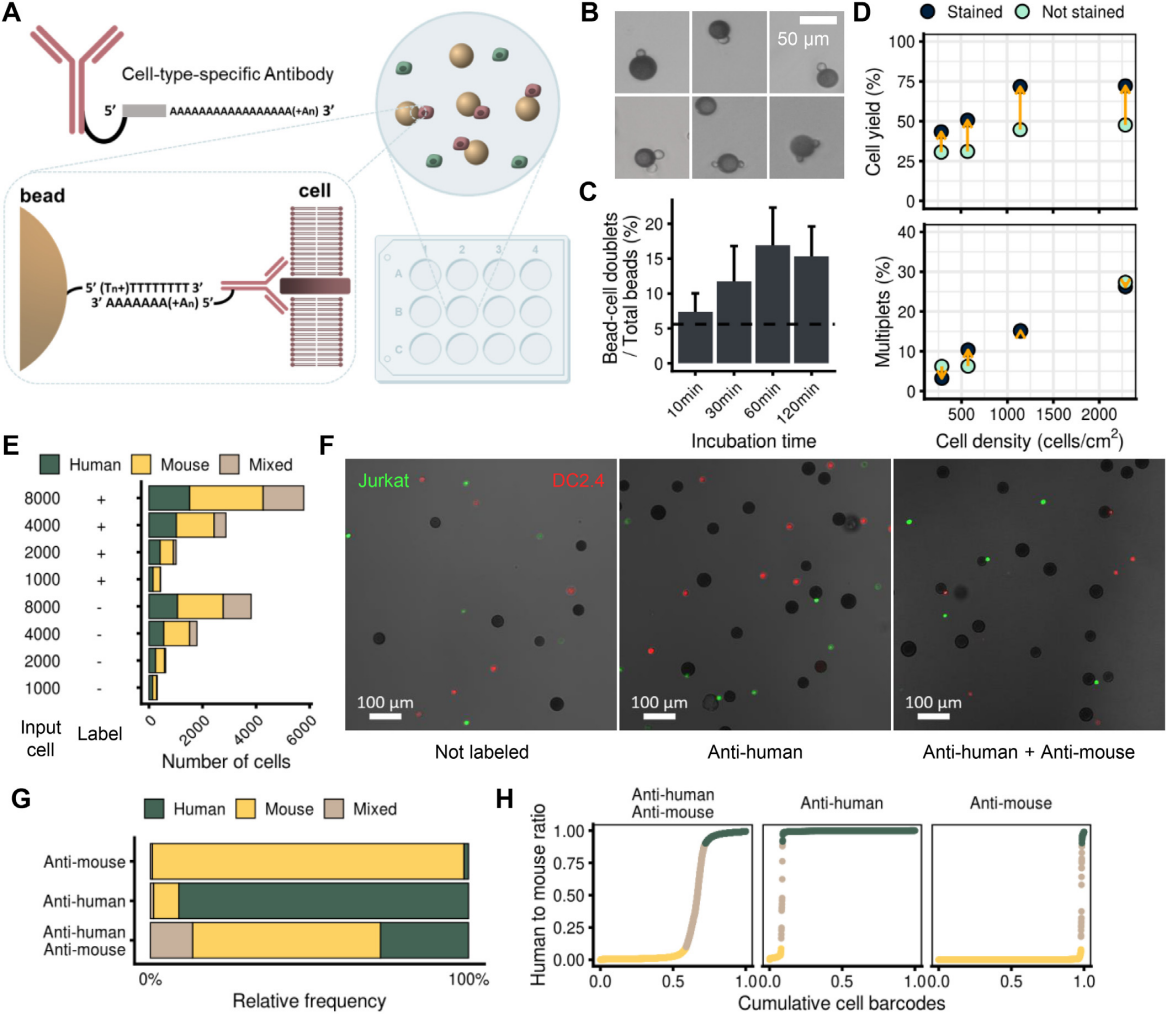
Development of aa-Onepot-Seq. (**A**) Scheme of aa-Onepot-Seq. Cells were labeled with a poly(A)-conjugated antibody and incubated with beads before lysis and processing by Onepot-Seq. The cell type targeted by the antibody preferentially formed a complex with beads, resulting in biased single-cell libraries consisting of the specific cell type. (**B**) Various types of bead–cell complexes. Bead–cell complexes (top, 1–3) and cell–bead–cell complexes (bottom, 4–6) are shown. (**C**) Formation of bead–cell complexes versus incubation time measured by FACS. The control group without incubation is indicated by a dashed line. (**D**) The effect of poly(A)-conjugated antibody labeling on cell yield (top) and the rate of multiplets (bottom) at various cell densities. Changes due to labeling are indicated by orange arrows. (**E**) The same experiment as (**D**), showing the number of cells and their identities for each sample. (**F**) Fluorescence micrographs with three types of antibody labeling: both human and mouse not labeled (left); labeled using only human-specific antibody (middle); and labeled using both human- and mouse-specific antibodies (right). The human cell line and mouse cell line were labeled with species-specific ‘Hashtag’ antibodies. (**G**) Selective capture of aa-Onepot-Seq. Relative frequencies of human and mouse cells that were sequenced after unbound cell depletion. Samples were labeled with only human-specific antibody, only mouse-specific antibody or both human- and mouse-specific antibodies. (**H**) The same experiment as (**G**), showing the human to mouse ratio along cell barcodes sorted in increasing order of human species identity.

Since Onepot-Seq uses a stochastic distribution of cells in a transient reaction chamber, we thought that increasing the affinity of the cell for the bead might increase the efficiency and the selectivity of the capture of single cells. Therefore, we devised aa-Onepot-Seq, which uses a poly(A)-conjugated antibody (32) to increase the affinity of the cell for the bead and to form the cell–bead complex via the formation of a poly(dT)–poly(A) hybrid **(**Figure 2A).

After incubating antibody-labeled cells with beads, we observed various types of cell–bead complexes including duplexes and triplexes (Figure 2B). We determined the complex formation versus incubation time via FACS and found the highest efficiency at 60 min (Figure 2C). We examined the effect of antibody labeling using different numbers of cells and obtained single-cell libraries under various conditions (Figure 2E). Comparative analysis between aa-Onepot-Seq and ordinary Onepot-Seq over varying cell densities revealed that labeling cells with poly(A)-conjugated antibody increased cell yield (Figure 2D, top), and did not affect the multiplet rate (Figure 2D, bottom).

Since antibodies are protein specific, we envisaged that aa-Onepot-Seq could capture a specific type of single cells. We observed cell type-specific cell–bead complexes using human- or mouse-specific antibodies (Figure 2F). In addition, we developed a washing protocol using a reversible cell strainer to remove unbound cells while recovering bound cells and beads, thereby increasing the selectivity of aa-Onepot-Seq (Supplementary Figures S4 and S5; see the Materials and Methods). Applying aa-Onepot-Seq with human- or mouse-specific antibodies to a mixed human–mouse population, we captured 98.5% of human cells and 92.4% of mouse cells, respectively, demonstrating the high selectivity of the method (Figure 2G). The ratio of human to mouse transcripts showed a high species specificity in each cell type, indicating high quality of the libraries (Figure 2H).

### Analysis of various cell types in PBMC populations using aa-Onepot-Seq

**Figure 3.**
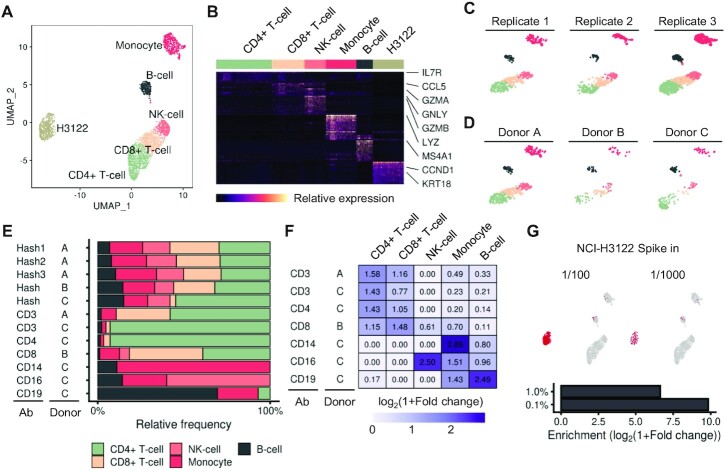
aa-Onepot-Seq used to profile targeted cell types in PBMCs using poly(A)-conjugated antibodies. (**A**) UMAP embedding of all samples from targeted sequencing of PBMCs. Cells are colored and labeled by their cell types. (**B**) A heatmap showing the expression of specific markers for each cell type. Some examples are labeled. (**C**) UMAP plot across three replicates from donor A. (**D**) UMAP plot across three donors. (**E**) Relative frequencies of cell types in single-cell libraries for each targeted antibody. The donor and antibody used for each sample are shown on the left side of the graph. (**F**) A heatmap showing the enrichment for each cell type for each sample. Fold change for the frequency of each cell type was calculated based on the composition of the ‘Hash’ sample from each donor. (**G**) Samples of PBMCs spiked with NCI-H3122 cells at 1/100 or 1/1000. Embedding of each sample in the UMAP plot, showing each major population as a distinct cluster different from other PBMC types (top). Enrichment of NCI-H3122 cells for each spike level compared with its original frequency (bottom).

To assess the ability of aa-Onepot-Seq to identify and enrich for specific cell types in a complex population, we used human PBMCs from three healthy donors with a variety of poly(A)-conjugated antibodies (Supplementary Figure S6; Supplementary Table S3). A total of 3845 single-cell libraries were obtained after filtering out cells with low-quality transcripts or low levels of transcripts. Unsupervised clustering analysis via UMAP (23) identified various subpopulations associated with unique expression markers (Figure 3A). The clusters were assigned to cell types based on the expression of known markers for major PBMC types (24) (Figure 3B; Supplementary Figure S7A). Three technical replicates using the ‘Hashtag’ antibody (32) that targets broadly expressed surface proteins in human tissues yielded similar subpopulation frequencies, demonstrating the reproducibility among different batches (Figure 3C). Projection of single cells from three different donors into UMAP space showed similar clustering patterns, indicating high reproducibility among biological replicates (Figure 3D). Each sample with poly(A)-conjugated antibodies targeting various immune CD markers showed a unique pattern both in cell type composition and UMAP space (Figure 3E; Supplementary Figure S7B). Fold changes for the frequency of each cell type in each sample were calculated based on the composition of the ‘Hash’ sample, which represents all the human cells from each donor. Specific cell types expressing the surface markers targeted by each poly(A)-conjugated antibody were enriched (Figure 3F). Specifically, monocytes were enriched 6.4-fold with the antibody targeting CD14, a surface protein marker mainly expressed by macrophages (33). Natural killer cells and B cells were most enriched in samples with CD16- and CD19-targeting antibodies, respectively. Anti-CD8 and anti-CD4 antibodies enriched their corresponding subtypes of T cells, although some overlaps reflected a discrepancy between transcriptomes and expressed proteins.

To assess the quality of the PBMC data of Onepot-Seq, we integrated the 10× chromium dataset from a public repository with our data and performed an integrative analysis of the two datasets. We observed comparable cell type proportions in both datasets, although the proportions differed slightly because the two datasets had different PBMC donors (Supplmentary Figure S15C, D, E, G). In addition, we performed an experiment using nuclei from PBMCs and compared the results with those of experiments using whole cells. We found that the cells and nuclei clustered well together and had comparable cell type compositions. Furthermore, the numbers of genes and transcripts captured per cell were comparable between the nuclei and the cells, showing the applicability of Onepot-Seq to the nucleus sample (Supplmentary Figure S15D, F, H)

CTCs are rare cells found in the blood of patients with solid tumors and are responsible for tumor metastasis (34–36). The frequencies of CTCs vary depending on tumor types and metastatic status, ranging from 1 to 10^4^ CTCs per 7.5 ml of blood (37,38). To mimic CTCs in a complex population in patients with advanced tumors, we spiked tumor cell lines to PBMCs from healthy donors. To selectively sequence CTCs, we used a poly(A)-conjugated antibody targeting epithelial cell adhesion molecule (EpCAM), a transmembrane glycoprotein expressed in most of the tumors of epithelial origin. Of the three cell lines tested, NCI-H3122 gave the best binding efficiency with beads and were used to mimic CTCs (Supplementary Figure S8). CTCs were added to PBMCs at two different levels of spike (1.0% and 0.1%) and sequenced using aa-Onepot-Seq. Tumor cells were identified by the formation of a cluster distinct from other cell types in PBMCs and the expression of epithelial markers such as KRT18 (Figure 3A, G). Tumor cells were enriched 99-fold and 913-fold in the 1.0% and 0.1% spiked samples, respectively. These results demonstrate the versatility of aa-Onepot-Seq, which can selectively capture and sequence rare cell types in a complex cell population.

### Drug treatments of multiple cell lines using Onepot-Seq

Gene expression profiling of chemical or genetic perturbations furnishes high content readouts, providing insights to elucidate a molecular portrait of underlying mechanisms (39–42). Recently developed methods for multiplexing samples for scRNA-seq using poly(A)-conjugated antibodies (43), barcoding oligos (44,45) and natural genetic barcodes (26) enabled large-scale screens of single-cell transcriptome profiling for pharmaceutical drug discovery by decreasing per sample cost.

**Figure 4.**
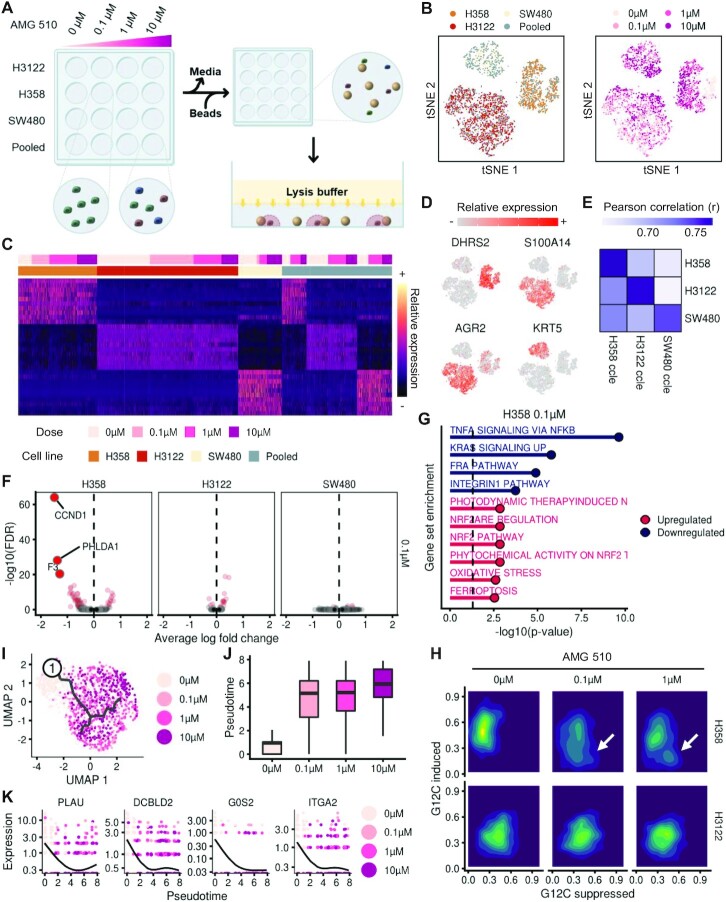
Dose-dependent AMG-510 drug perturbation of three cell lines each with a different KRAS mutation status. (**A**) Scheme of the experiment. Each cell line was treated with AMG-510 for 6 h at various doses and subjected to Onepot-Seq after removing the medium and adding beads to the wells. Pooled samples in which all three cell lines were mixed together are also included. (**B**) *t*-SNE plot colored by cell line identity (left) and dose (right). (**C**) A heatmap showing the expression of cell line-specific markers for single cells sorted by cell line identity and dose. Note that single-cell identities expressing specific cell line markers were preserved in the pooled samples. (**D**) Relative expression of representative genes expressed in specific cell lines. (**E**) Correlation between the average expression of each cell line in this experiment and the bulk expression data from CCLE. (**F**) Volcano plot showing DEGs for each cell line at 0.1 μM drug dose compared with the control. Genes that have a *P*-value < 0.05 and an absolute value of log (fold change) > 0.25 are colored red. (**G**) Gene set enrichment analysis using genes differentially expressed by AMG-510 treatment at 0.1 μM in H358 cells. (**H**) The distribution of KRAS(G12C) output scores across single cells. White arrows indicate single-cell populations that occurred after AMG-510 treatment in the H358 cell line with the G12C mutation. (**I**) UMAP plot of the H358 cell line and a dose–response curve with a root node chosen from untreated cells as the origin. (**J**) A boxplot showing the distribution of pseudotimes for single cells at each dose. (**K**) Representative genes that varied with pseudotime. Cells are colored by the treatment doses.

Onepot-Seq is ideal for automation and high-throughput screens since samples can be projected to sequencing directly without sample processing. As a proof of concept, we profiled three cancer cell lines with different oncogenic KRAS mutations by treatment with AMG-510 at various doses **(**Figure 4A). AMG-510 is an FDA-approved covalent inhibitor of KRAS(G12C) that irreversibly locks it in an inactive GDP-bound state (46,47). To capture the mutation-specific responses of AMG-510, NCI-H358, NCI-H3122 and SW480 with KRAS, genotypes of G12C, G12V and the wild type were selected. After AMG-510 treatment of cells, the medium was removed and beads were added in the same well, avoiding the need for additional sample processing before samples were analyzed using the Onepot-Seq protocol (see the Materials and Methods). Pools of all three cell lines in the same well were also included to verify the feasibility of sample multiplexing (Figure 4A). After sequencing and filtering out low-quality cells, the transcriptomes for 3949 single cells identified three distinct clusters corresponding to each cell line (Figure 4B). Each cell line corresponded to a unique cluster, while pooled samples comprised three clusters (Figure 4B, left). Each cell line showed a unique distribution for each of the four AMG-510 doses within each cluster (Figure 4B, right). Notably, NCI-H358, a KRAS(G12C) cell line, showed a clear shift in two-dimensional space upon treatment, reflecting KRAS(G12C)-specific inhibition by AMG-510. However, the cell cycle states of single cells did not vary upon treatment (Supplementary Figure S9). Each cell line exhibited a unique expression pattern with its own markers, independent of the AMG-510 dose (Figure 4C, D). Specifically, single cells in the pooled samples exhibited the unique expression signature for each cell line, demonstrating the preservation of single-cell identities while pooling (Figure 4C). The average gene expression for each cell line showed the highest correlation with the corresponding bulk expression data from the Cancer Cell Line Encyclopedia (CCLE) database, confirming the identification of cell lines (Figure 4E). An SNP-based classification of cell lines also agreed with the transcriptome-based classification, suggesting the applicability to SNP-based multiplexing (26) (Supplementary Figure S10).

For each KRAS cell line, we identified genes that were differentially expressed compared with controls for each AMG-510 dose. Responses were highly dependent on KRAS mutations and were highest in the KRAS(G12C) cell line (Figure 4F; Supplementary Figures S11 and S12). Gene set enrichment analysis of AMG-510-induced genes for each cell line revealed that KRAS and related pathways were down-regulated (Figure 4G) in the KRAS(G12C) cell line while genes associated with cell stress were up-regulated in non-KRAS(G12C) cell lines (Supplementary Figure S13). We used previously identified KRAS(G12C)-dependent genes (40) to derive G12C-induced and G12C-suppressed scores as indicators of the KRAS signaling output for each single cell (see the Materials and Methods). AMG-510 treatment decreased the G12C-induced score and increased the G12C-suppressed score in the KRAS(G12C) cell line even at low doses, while it did not change the scores in non-KRAS(G12C) cell lines (Figure 4H; Supplementary Figure S10C). To capture the continuously varying gene expression patterns in the KRAS(G12C) cell line, we performed pseudotime analysis and ordered cells along a trajectory by defining the node closest to untreated cells as the origin (Figure 4I). Pseudotimes of single cells exhibited a binary pattern depending on the treatment rather than a continuum across the four doses (Figure 4J). However, continuously varying modules were identified, suggesting a heterogeneous drug response within each treated sample. Genes regulated by KRAS such as PLAU, DCBLD2 and ITGA2 were down-regulated across pseudotime (Figure 4K; Supplementary Figure S14). Taken together, these results demonstrate that Onepot-Seq captures drug-induced responses in gene expression and the responses contributed by the underlying pathways.

## DISCUSSION

Here we present Onepot-Seq, a simple and routine platform for single-cell transcriptome analysis. Onepot-Seq uses localized diffusion of mRNA within sparsely distributed cells in one pot. We used Poisson's model to build the theoretical foundation for Onepot-Seq and verified it through multiple types of experiments. To increase the capture efficiency and selectivity, we devised aa-Onepot-Seq, which selectively captures specific cell types with poly(A)-conjugated antibodies. Lastly, we applied Onepot-Seq to profile dose-dependent responses of cancer cell lines to the KRAS(G12C) inhibitor AMG-510 and observed G12C mutation-specific responses of KRAS-dependent genes at single-cell resolution.

Onepot-Seq provides a variety of advantages over conventional scRNA-seq. The simultaneous capture of mRNA from thousands of single cells in one pot simplifies library construction and reduces the time, labor and overall costs for single-cell profiling. Onepot-Seq eliminates the need for single-cell compartmentalization, which requires sophisticated instruments, hence lowering the barrier to entry of scRNA-seq in various laboratories. The technique is applicable when working with low input samples with limited sources; it does not require sample processing with the associated sample losses. Lastly, samples can be sequenced directly after treatment, preserving dynamic transcriptional variations. We also performed a cost analysis comparing the reagents and barcoded beads used in Onepot-Seq with those used in Drop-Seq, showing that Onepot-Seq is advantageous when processing with a small number of samples (Supplementary Table S4).

However, Onepot-Seq still has some limitations. Because it utilizes a transient reaction chamber, it can be highly sensitive to ambient RNA contamination from apoptosis or damaged cells, such as drug-treated cells. Therefore, caution may be required when treating samples with high ratios of such cells. The use of fresh cells and washing to remove ambient RNA before lysis can minimize these effects. As the cost of sequencing decreases, the demand for scRNA-seq is growing. However, technological advances in scRNA-seq have not kept pace with the growth in demand associated with advances in sequencing. Onepot-Seq is easily customizable and optimal for automation, providing a simple single-cell platform that can be used to screen cells subjected to a variety of perturbations. Onepot-Seq may thus help bridge the gap between the decreasing cost of sequencing and the high cost of single-cell library construction, thereby contributing to the widespread adoption of scRNA-seq.

## DATA AVAILABILITY

Full sequencing data were deposited in the Sequence Read Archive (PRJNA748601). All data generated or analyzed during this study are included in the main text or the supplementary data. Additional data related to this paper will be provided by the corresponding authors upon request.

## Supplementary Material

gkac665_Supplemental_Files
