# Supplementary material for: Onepot-Seq: capturing single-cell transcriptomes simultaneously in a continuous medium via transient localization of mRNA
Source: Nucleic Acids Res. 2022 Aug 12;50(22):12621–35. doi: 10.1093/nar/gkac665 (PMC9825186; doi:10.1093/nar/gkac665)
Supplement: gkac665_Supplemental_Files [file gkac665_supplemental_files.zip › Onepot-Seq_NAR_supplementary figures.pdf]

Supplementary Figures

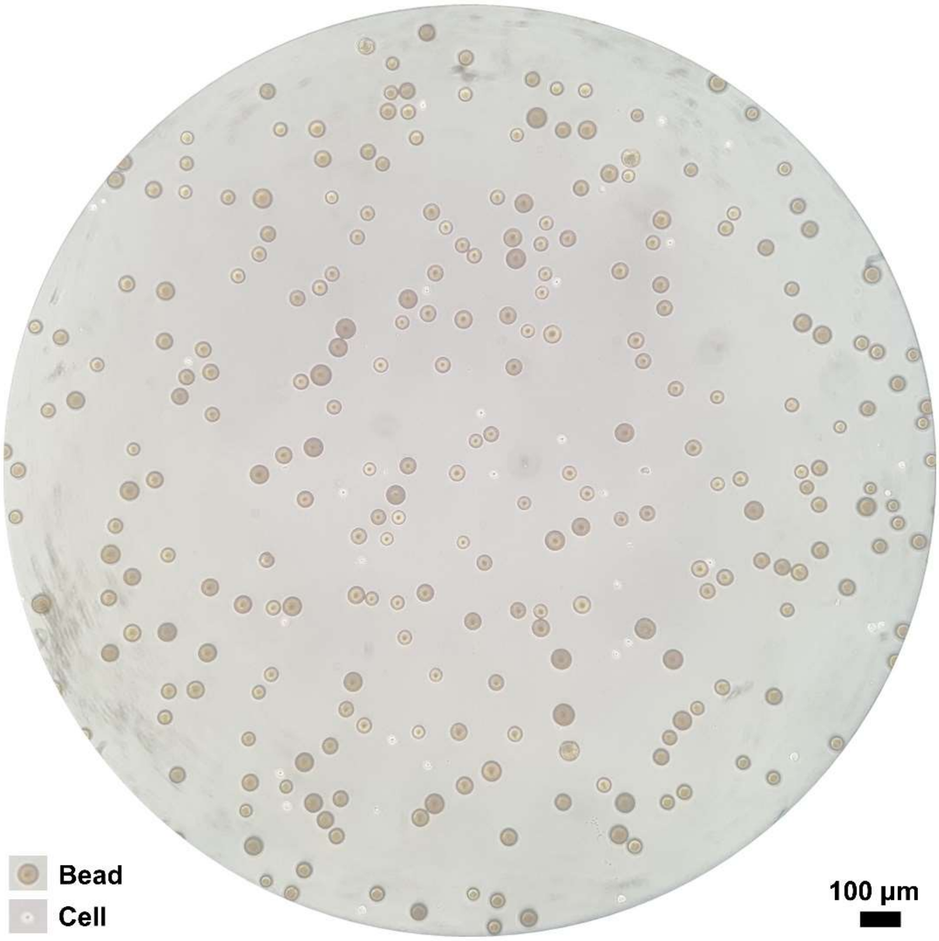

Supplementary Figure S1. A micrograph showing the distribution of beads and cells in Onepot-Seq.

20,000 beads and 2,000 cells were added to a well of a 12-well plate and gently rocked three times vertically and three times horizontally to evenly spread beads and cells. Beads and cells were incubated for 15 min before imaging. The number of cells within the radius re of the beads follows a Poisson distribution.

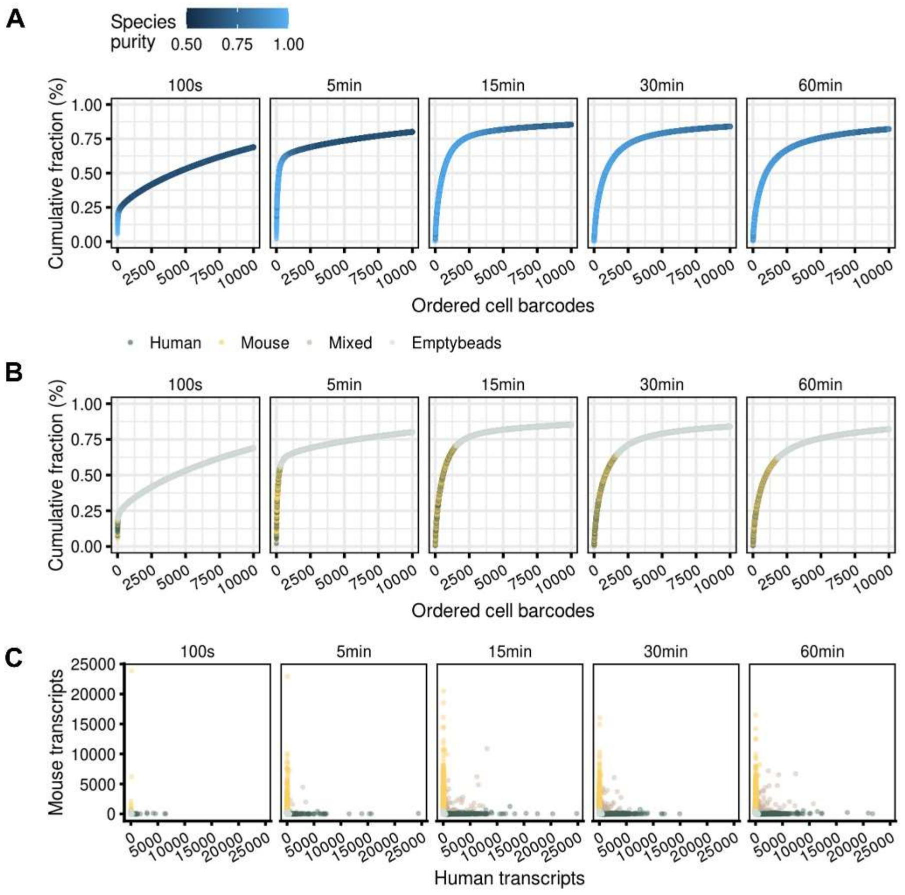

Supplementary Figure S2. Analysis of the species-mixing experiment with various times of lysis.

(**A**) Curve plots showing the cumulative fraction of reads sorted by decreasing order of the size of cell barcodes (number of reads) colored by species purity. The species purity of cell barcodes drops dramatically after the inflection point of each curve, showing that the barcodes after the inflection point correspond to the beads that captured the ambient RNA. Note that at 15 min of lysis, the curve shows an inflection point and reaches maximum cell capture. (**B**) Same as (**A**) except the plot is colored by cell identity. (**C**) Scatter plot showing the number of human and mouse transcripts associated with each cell barcode.

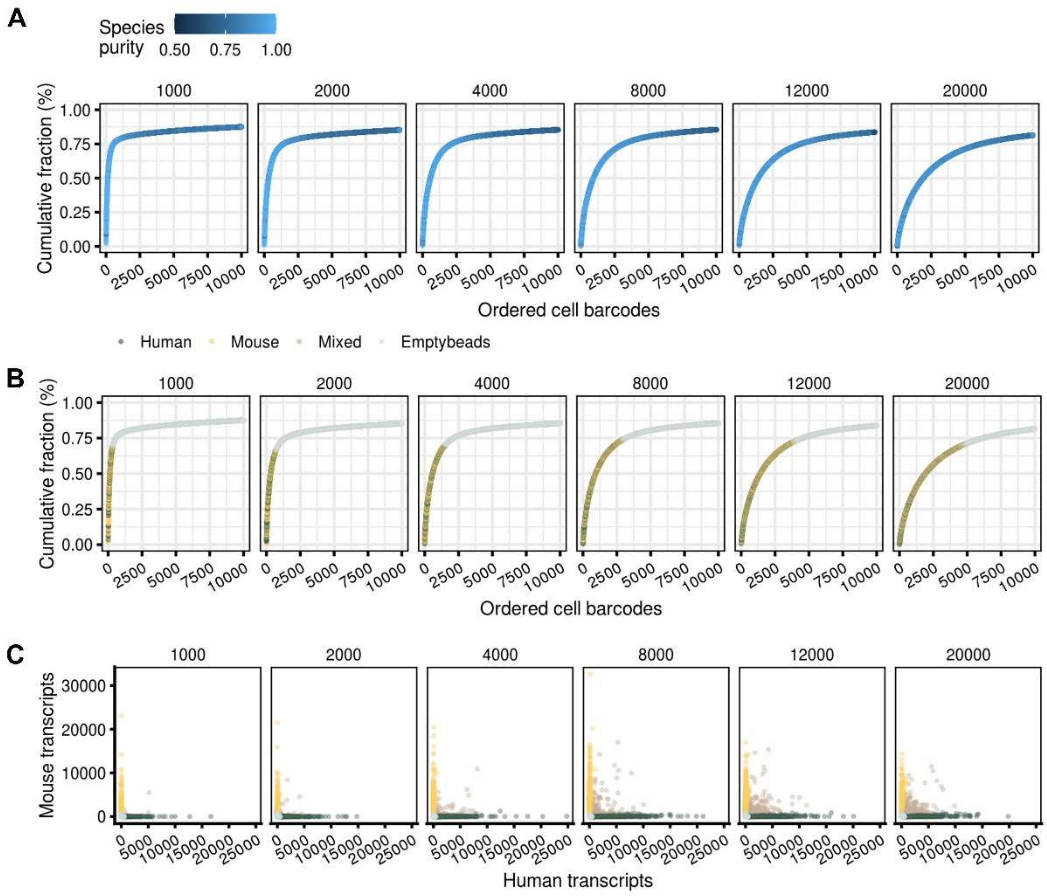

Supplementary Figure S3. Analysis of the species-mixing experiment with various cell densities.

Same as Extended Data Figure S2 except that samples with various cell densities were analyzed. (**A**) Curve plots showing the cumulative fraction of reads sorted by decreasing order of the size of cell barcodes (number of reads) colored by species purity. (**B**) Same as (**A**) except the plot is colored by cell identity. (**C**) Scatter plot of samples showing the number of human and mouse transcripts associated with each cell barcode.

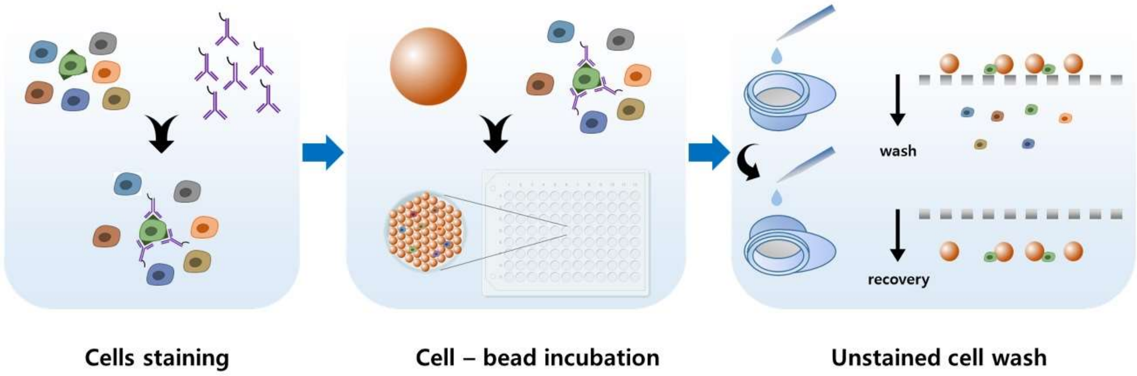

Supplementary Figure S4. Scheme of the experiment showing the depletion of unbound beads using an invertible strainer in aa-Onepot-Seq.

Scheme of aa-Onepot-Seq and depletion of unbound cells: (1) Cells were labeled with poly(A)-conjugated antibodies that target markers expressed by the cells. (2) Labeled cells were incubated with washed beads in a well of 96-well plate to maximize cell-bead contact. (3) After incubation, cells and beads were transferred to the invertible cell filter with a mesh size larger than the cells but smaller than the beads. (4) Unbound cells were washed away by adding and swirling the wash buffer three times. (5) After the final wash, the filter was inverted and the remaining cells and beads on the strainer were recovered by adding buffer to the strainer.

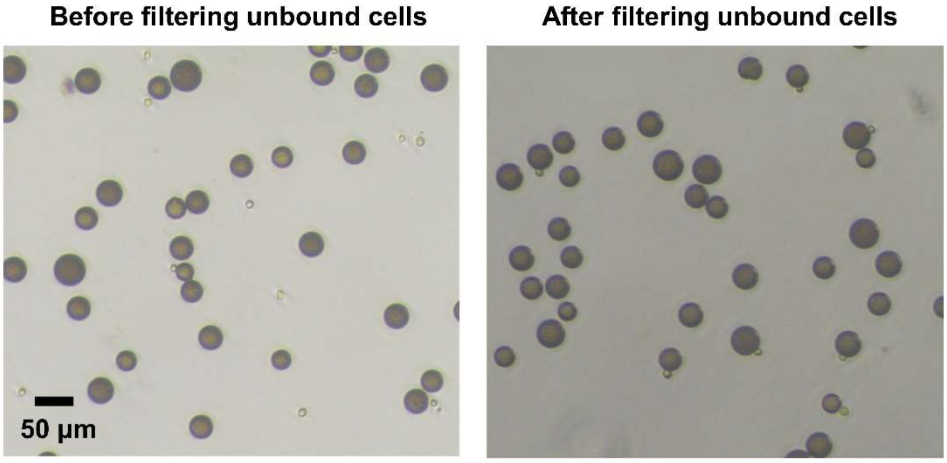

Supplementary Figure S5. Micrographs showing the distribution of cells and beads before and after unbound cell filtering.

Jurkat and DC2.4 cells were labeled with anti-human and anti-mouse poly(A)-conjugated antibodies, respectively. Jurkat and DC2.4 cells were mixed before incubation with beads. Cells were incubated with beads for 1 hour before imaging. Unbound cells were then filtered and the distribution of beads and cells before filtering unbound cells (left) and after filtering unbound cells (right) was determined. Note that unbound cells were clearly removed by the filtering.

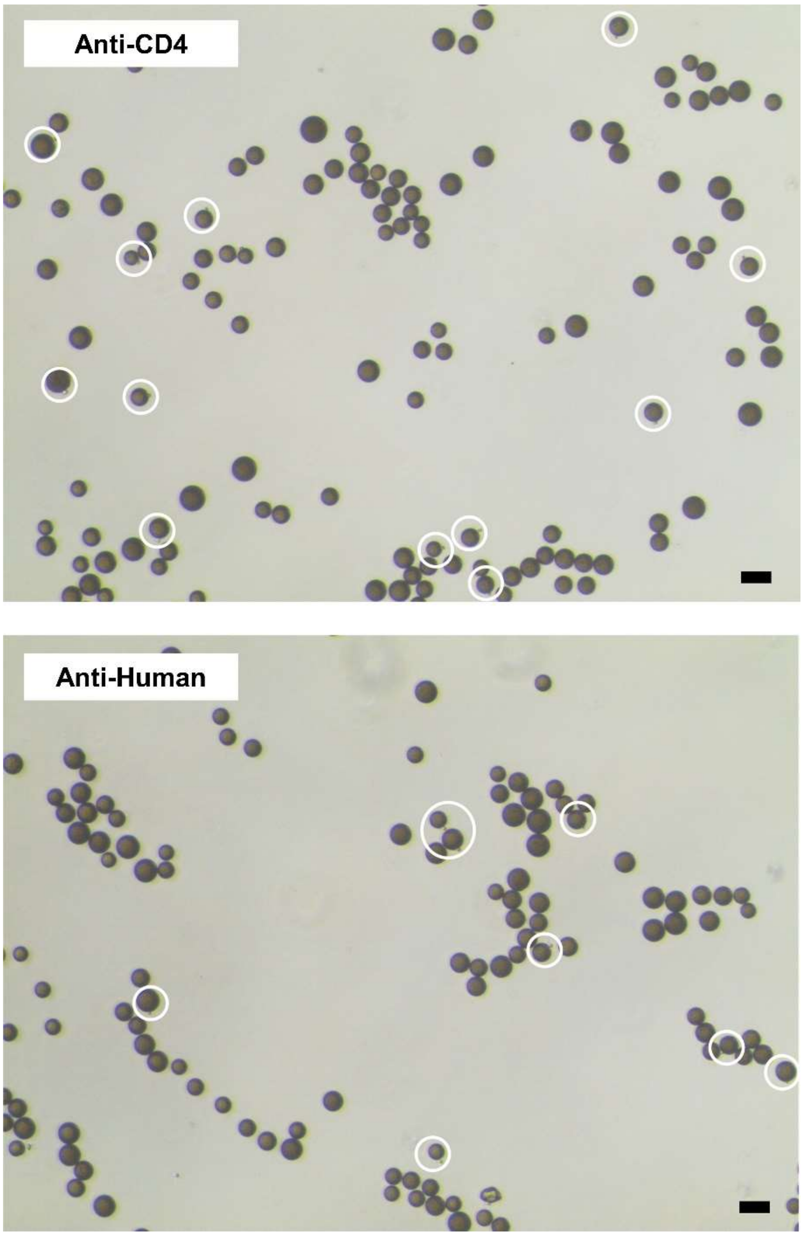

Supplementary Figure S6. Micrographs showing cell-bead complex formation in PBMC samples.

Human PBMCs were labeled with poly(A)-conjugated antibodies that target various CD markers and specifically capture unique cell types. Two representative samples, anti-CD4 (top) and anti-human (bottom) are shown. Cells were labeled, incubated with beads, and unbound cells were removed by cell filtering before imaging. Cell-bead complexes are indicated with white circles.

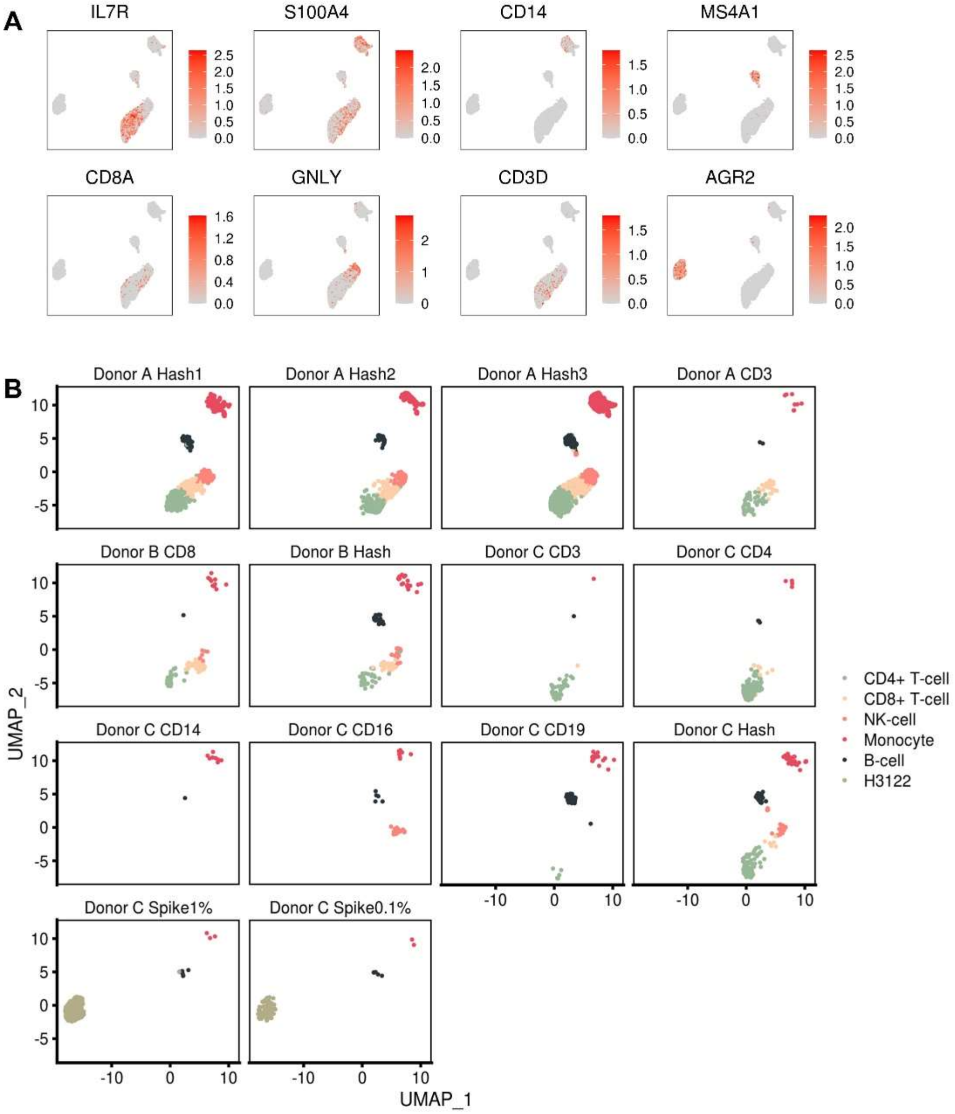

Supplementary Figure S7. The expression of cell type markers and the cell type compositions of samples in PBMC experiment

(**A**) Relative expression of cell type-specific markers in PBMC samples. (**B**) UMAP plots that show the compositions of cell types in each sample. Cells were colored by their cell types.

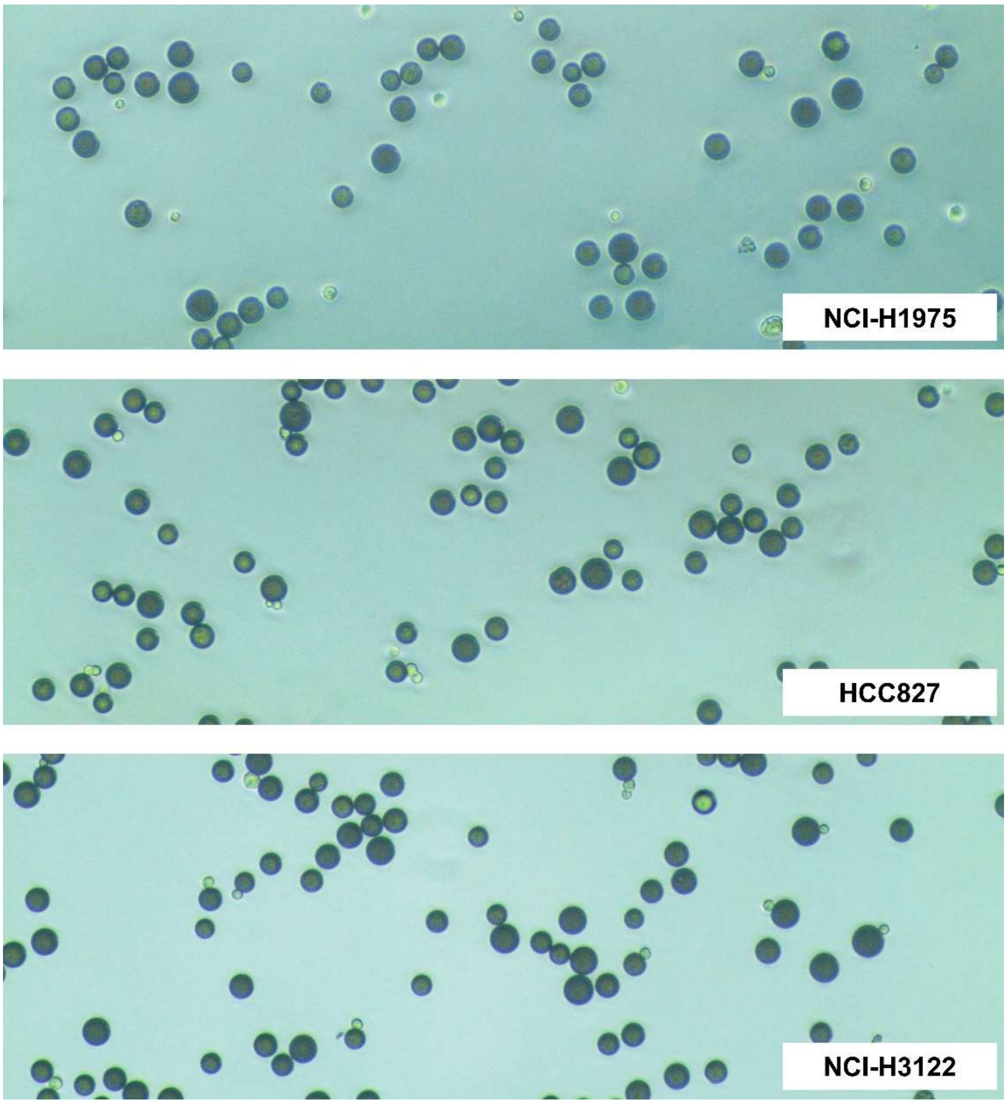

Supplementary Figure S8. Micrographs showing the efficiencies of cell-bead complex formation in various cancer cell lines using an EpCAM antibody.

To mimic CTCs from cancer patients, three cancer cell lines that are known to express EpCAM were tested for cell-bead complex formation efficiency after labeling with anti-EpCAM and incubating with beads. Cells were incubated with beads in a well of 96-well plate for 1 hour and transferred to a well of a 12-well plate before imaging. NCI-H1975 (top), HCC-827 (middle), and NCI-H3122 (bottom).

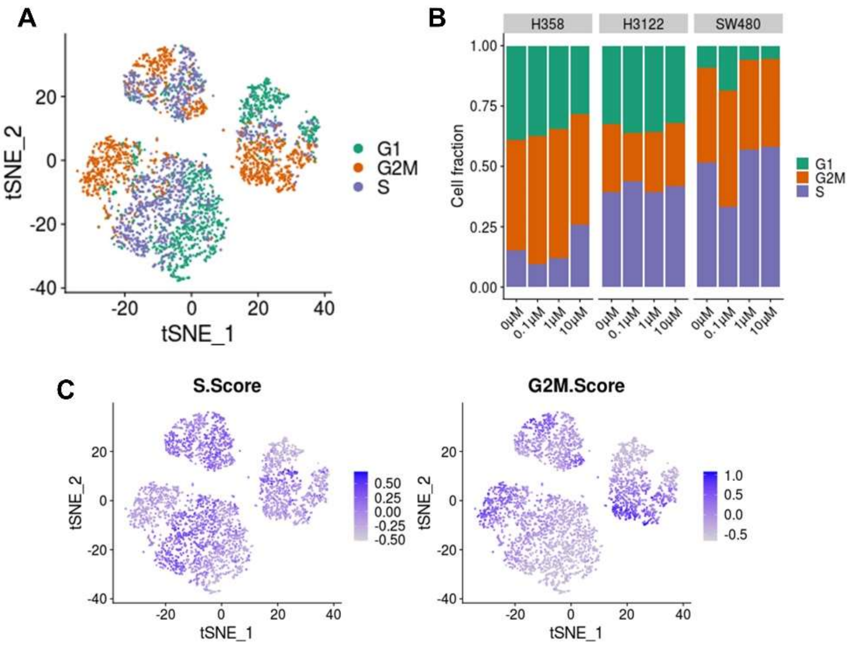

Supplementary Figure S9. Cell cycle analysis of dose-dependent AMG-510 treated cells.

Cells were classified to G2-M, S, or G1 phase using reported cell cycle markers and cell cycle score calculations (see Methods). (**A**) tSNE plot of single cells colored by cell cycle states. (**B**) Relative frequencies of cell cycle states for each sample. Note that the composition of cell cycle states does not dramatically change with drug treatment. (**C**) tSNE plots of single cells are colored by S.Score (left) and G2M.score (right).

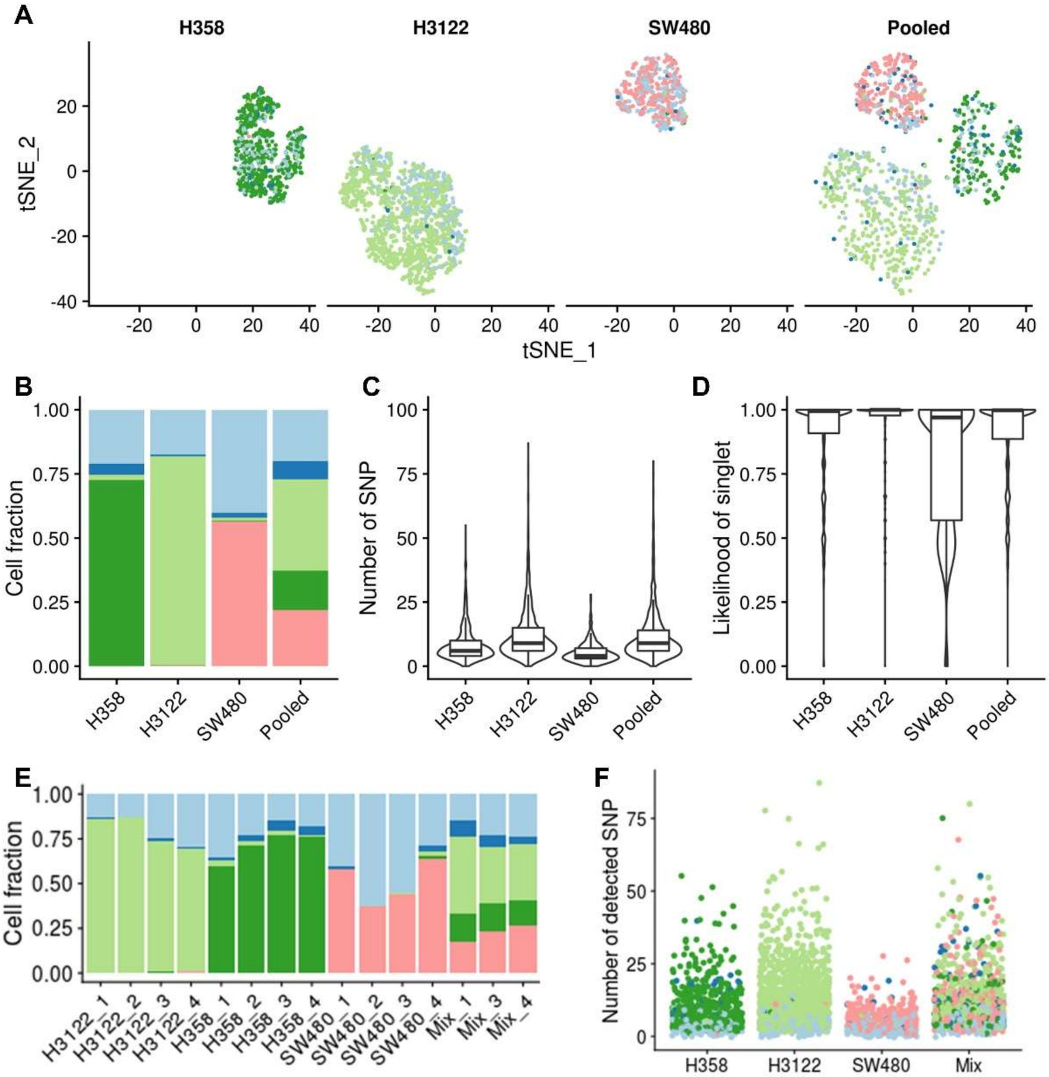

Supplementary Figure S10. SNP-based classification of cell lines using demuxlet

Results of cell line classification by demuxlet using SNPs of single cells. (**A**) tSNE plots of each sample are colored by the classification results. Note that in the "pooled" sample, the classification based on the gene expression (coordinates in tSNE plot) is consistent with the classification based on SNPs, indicating highly specific single-cell libraries in Onepot-Seq. (**B**) Relative frequencies of cell line classification in each cell line sample. Unknown cells are due to the small number of SNPs in the cells. (**C**) Violin plot showing the number of SNPs in single cells. (**D**) Likelihood of singlet for single cells obtained from demuxlet results. (**E**

) Relative frequencies of cell line classifications in each sample. (**F**) Scatterplot showing the number of SNPs for each sample colored by the classification results.

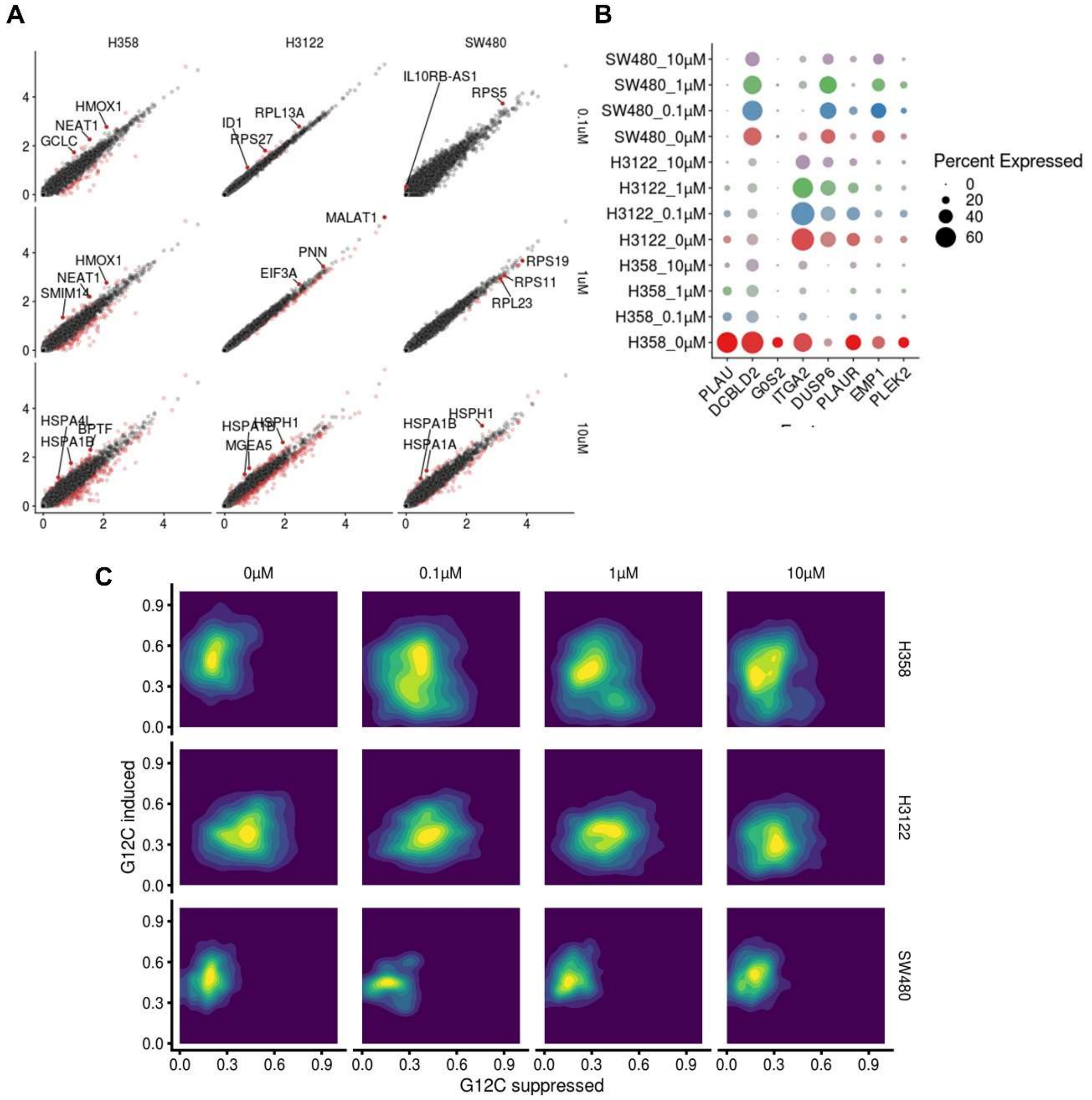

Supplementary Figure S11. Analysis of transcriptional responses of three cell lines with AMG-510 treatment.

(**A**) Scatter plot showing the bulk-averaged gene expression of each sample compared to the control (untreated) sample. Genes that have a P-value < 0.05 and an absolute value of log (fold change) larger than 0.25 are colored red. (**B**) Dot plot showing G12C-specific changes in gene expression. The size of the dot corresponds to the percentage of cells expressing the change in each cluster. The intensity of the color represents the average expression level. Note that only H358 cells with the G12C mutation responded in expression after AMG-510 treatment. (**C**) The distribution of KRAS(G12C) output scores across single cells for each sample.

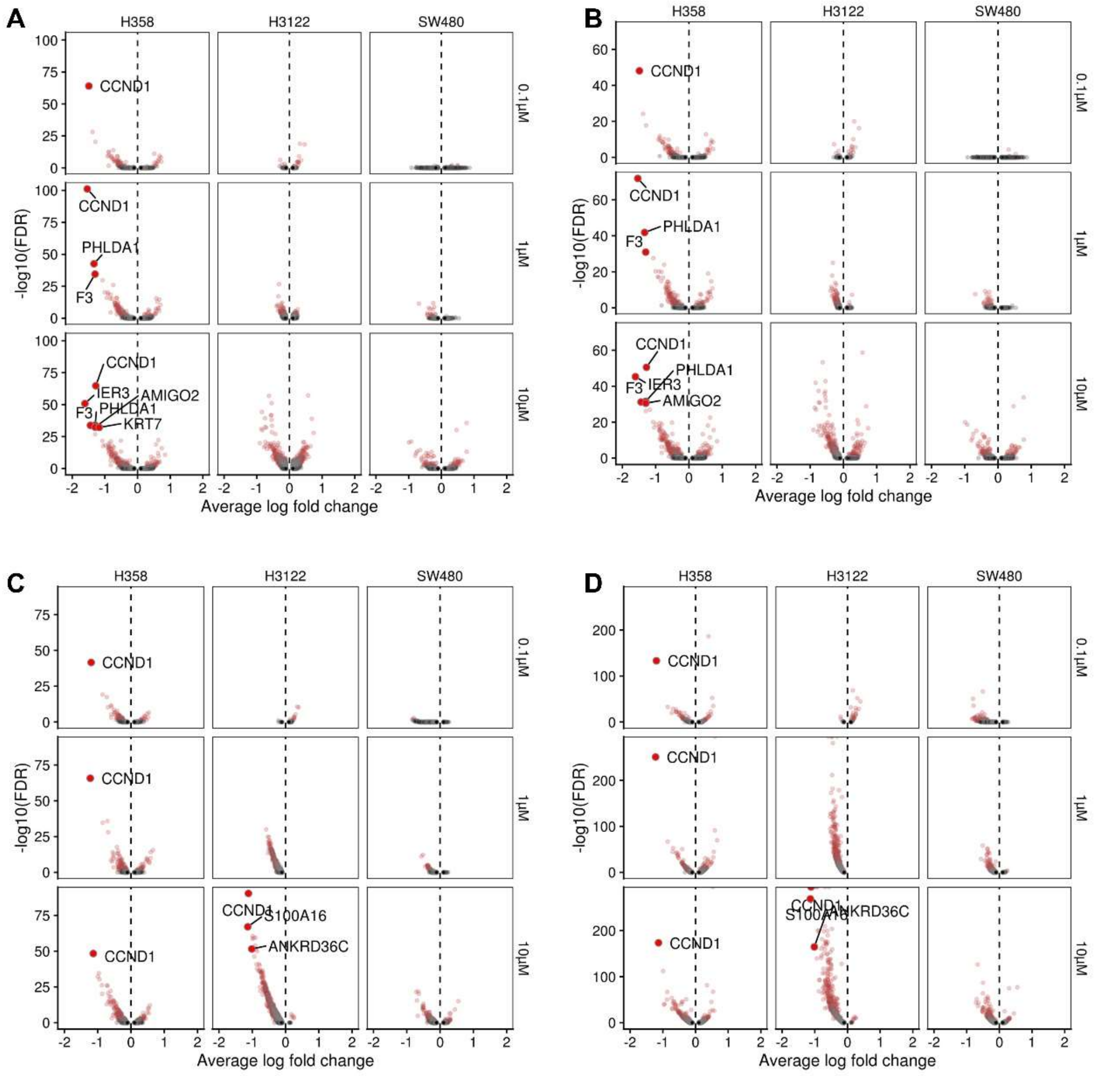

Supplementary Figure S12. Volcano plots showing differentially expressed genes.

Volcano plots showing differentially expressed genes for each cell line at each drug dose compared with the control. Various tests were used for the plots. Genes that have a P-value < 0.05 and an absolute value of log (fold change) larger than 0.25 are colored red. (**A**) Volcano plots using a likelihood-ratio test. (**B**) Volcano plots using Wilcoxon rank-sum test. (**C**) Volcano plots using a negative binomial generalized linear model. (**D**) Volcano plots using a Poisson generalized linear model.

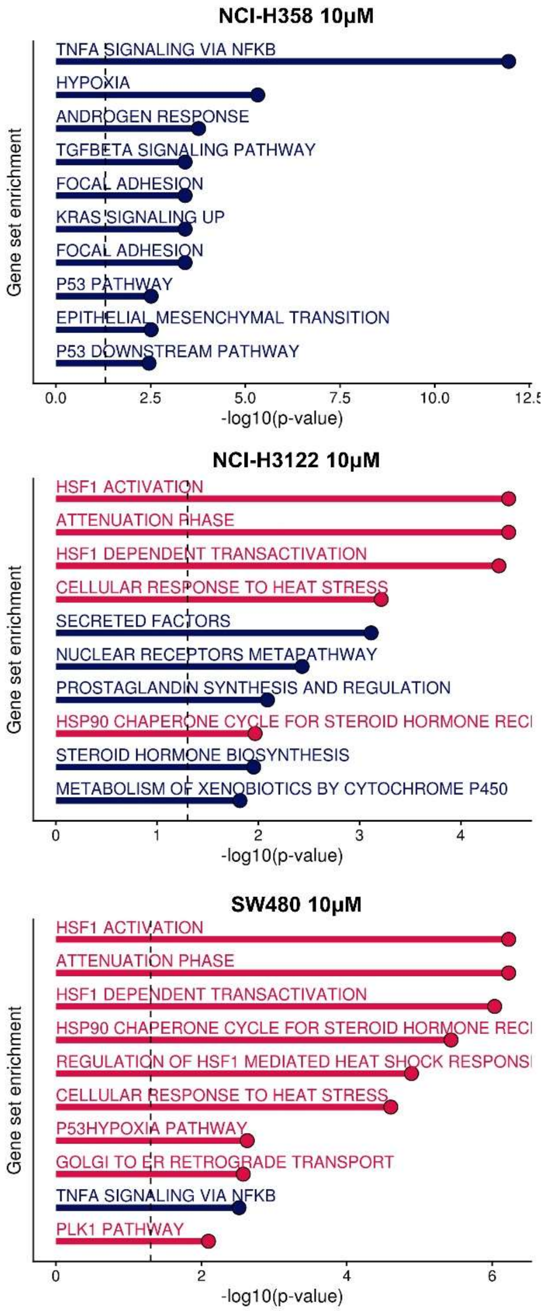

Supplementary Figure S13. Gene set enrichment analysis of three cell lines with AMG-510 treatment at 10 µM dose

Gene set enrichment analysis using genes differentially expressed by AMG-510 treatment at 10 µM dose in NCI-H358 (top), NCI-H3122 (middle), and SW480 (bottom) cell lines. Note that NCI-H358 cell line enriched genes with KRAS and related pathways while NCI-H3122 and SW480, which are non-KRAS(G12C) cell lines, are associated with pathways related to the cell stress.

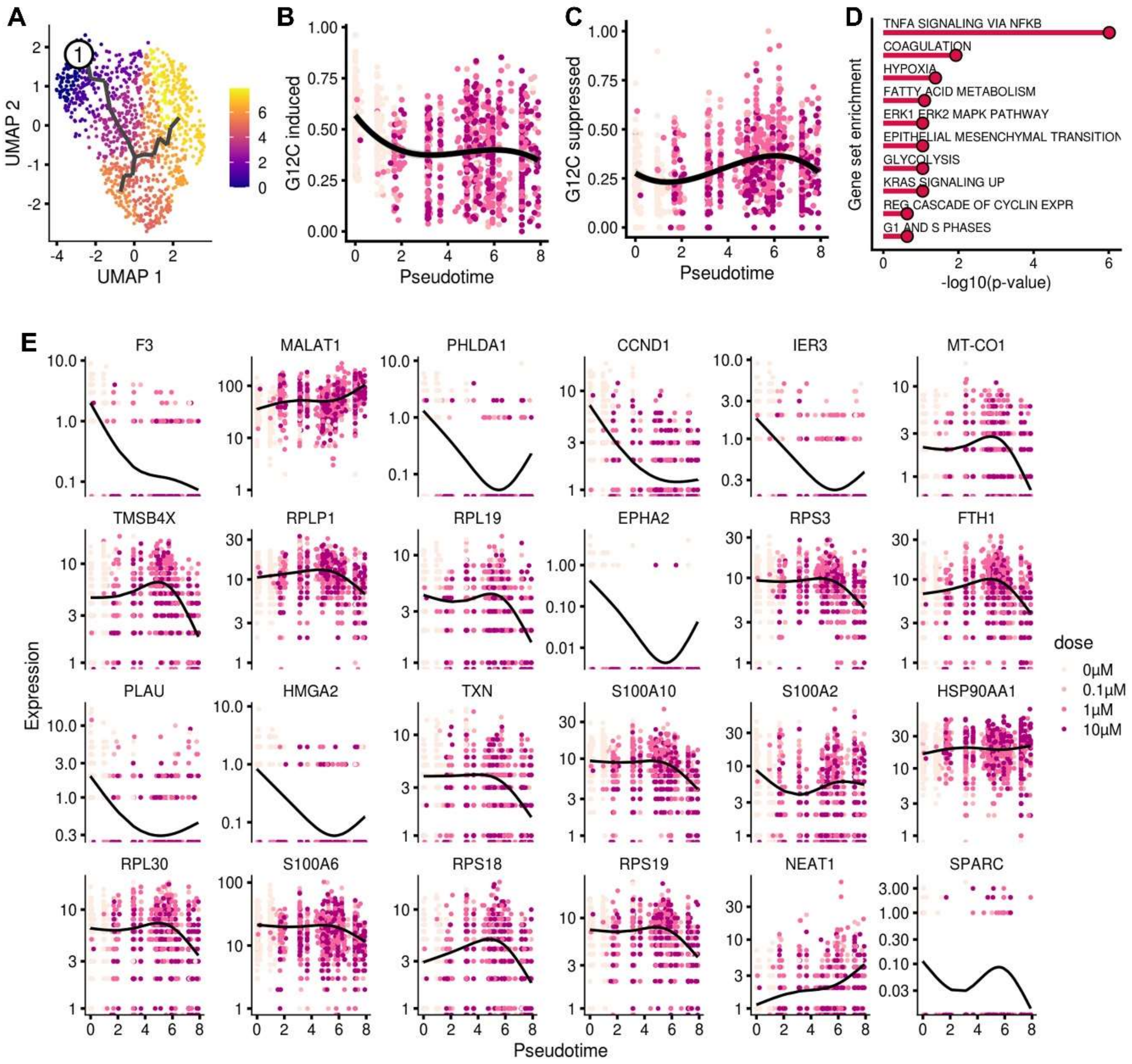

Supplementary Figure S14. Pseudotime analysis of AMG-510 treatment in NCI-H358 cells.

(**A**) UMAP plot of H358 cells and a dose-response trajectory colored by pseudotime. The root node was chosen from untreated cells. (**B**) The G12C-induced score of single cells as a function of pseudotime. (**C**) The G12C-suppressed score of single cells as a function of pseudotime. (**D**) Gene set enrichment analysis using the top 42 variable genes along pseudotime after AMG-510 treatment in H358 cells. (**E**) Gene expression patterns of single cells colored by drug doses as a function of pseudotime. The top 24 DEGs in descending order of q value that were used in creating the plots.

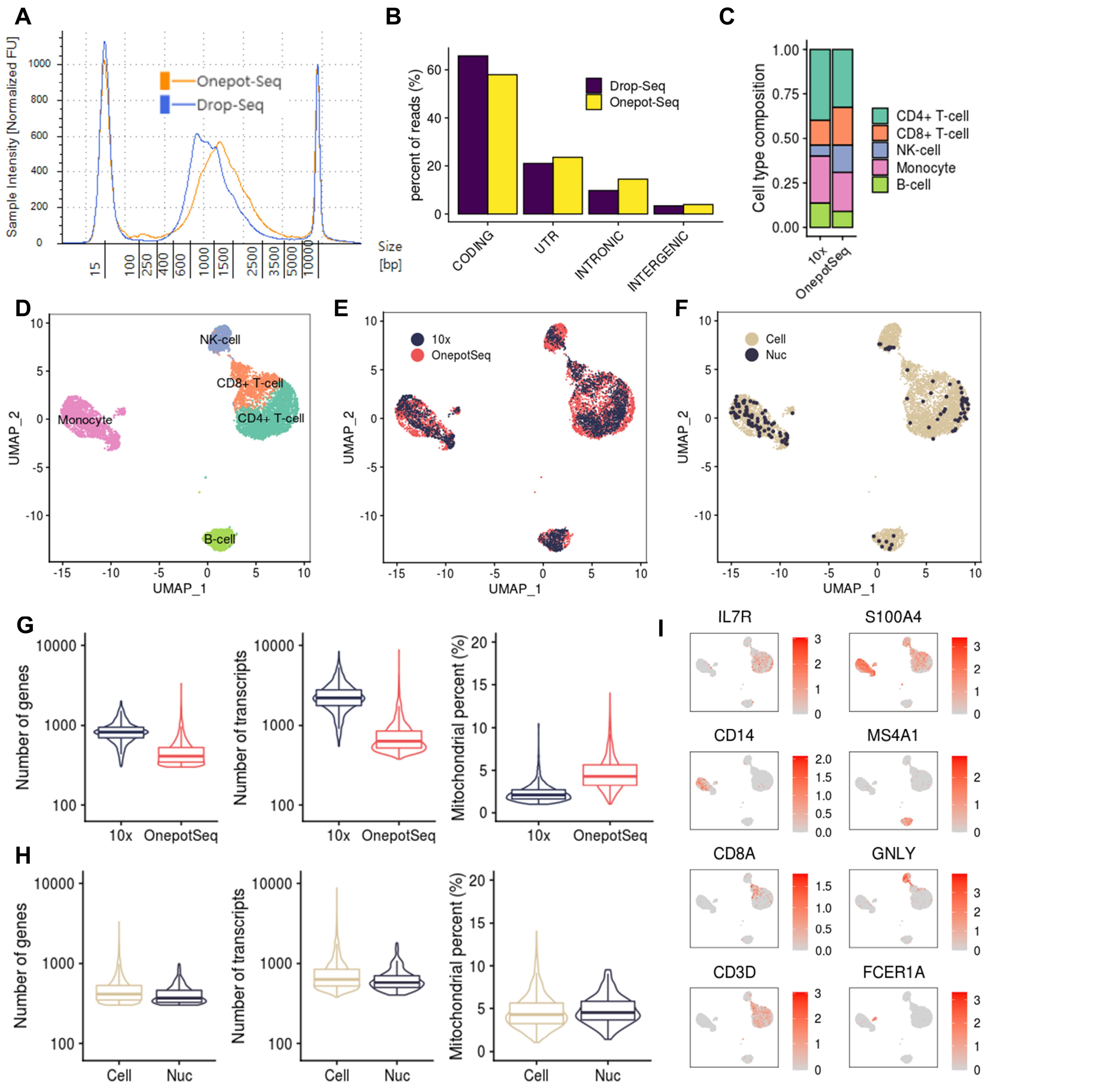

Supplementary Figure S15. Systematic comparison of Onepot-Seq with other scRNA-seq platforms and the applicability of Onepot-Seq to nucleus sample.

(**A**) cDNA traces of Onepot-Seq and Drop-Seq libraries made with HSD1000 TapeStation, showing the length distribution of the cDNA libraries. (**B**) Percent genome-mapped reads mapped to coding regions, UTRs, introns, and intergenic regions for Onepot-Seq and Drop-Seq. (**C**) Relative frequencies of cell types in single-cell data of PBMCs for Onepot-Seq and the 10x public repository dataset. (**D**) UMAP embedding of all samples from the 10x and Onepot-Seq datasets. (**E**) UMAP plot colored by dataset (10x and Onepot-Seq). (**F**) UMAP plot colored by cell and nucleus for Onepot-Seq. (**G**) Violin plot showing the number of genes (left), number of transcripts (middle), and percentage mitochondrial genes (right) per cell for the 10x and Onepot-Seq datasets. (**H**) Violin plot showing the number of genes (left), number of transcripts (middle), and percentage mitochondrial genes (right) per cell for cells and nuclei. (**I**) UMAP plots showing the relative expression of representative genes expressed in PBMC single-cell data.

Supplementary Table S1. Differentially expressed genes for AMG-510 treatment per dose per cell line

Supplementary Table S2. Gene set enrichment analysis for AMG-510 treatment per dose per cell line

Supplementary Table S3. Statistics of the aa-Onepot-Seq experiments

Supplementary Table S4. Cost analysis of Drop-seq, Onepot-seq, and aa-Onepot-seq for various numbers of cells and samples
